# AdventML: Advanced Enzyme Temperature Prediction with Transformer-Based Embeddings and Resampling Strategies

**DOI:** 10.1021/acs.jcim.6c02109

**Published:** 2026-10-12

**Authors:** Jaldert François, Bart De Moor, Vera van Noort

**Affiliations:** † Centre of Microbial and Plant Genetics (CMPG), 26657 KU Leuven, 3001 Leuven, Belgium; ‡ Department of Electrical Engineering (ESAT), KU Leuven, 3001 Leuven, Belgium; § Institute of Biology Leiden (IBL), Leiden University, 2333 BE Leiden, The Netherlands

## Abstract

Accurate prediction of enzyme optimal catalytic temperature (*T*_opt_) is important for enzyme applications, particularly for identifying biocatalysts stable in extreme conditions, but experimental measurement remains costly and time-consuming. Here, we present AdventML, a machine learning framework for (*T*_opt_) prediction that expands the available models, evaluates protein representations and model architectures, and compares resampling strategies for improving performance at extremes. The base AdventML model achieved the best global held-out performance among the evaluated methods (*R* ^2^ ≈ 0.64), MAE ≈7–8 °C, while tail-focused variants improved selected extreme-temperature regimes, reducing tail MAE by up to ∼1.8 °C. AdventML also showed that competitive (*T*_opt_) prediction is possible in the absence of organism growth temperature. Our *T*_opt_ prediction models are made freely available as AdventML on GitHub.

## Graphical abstract

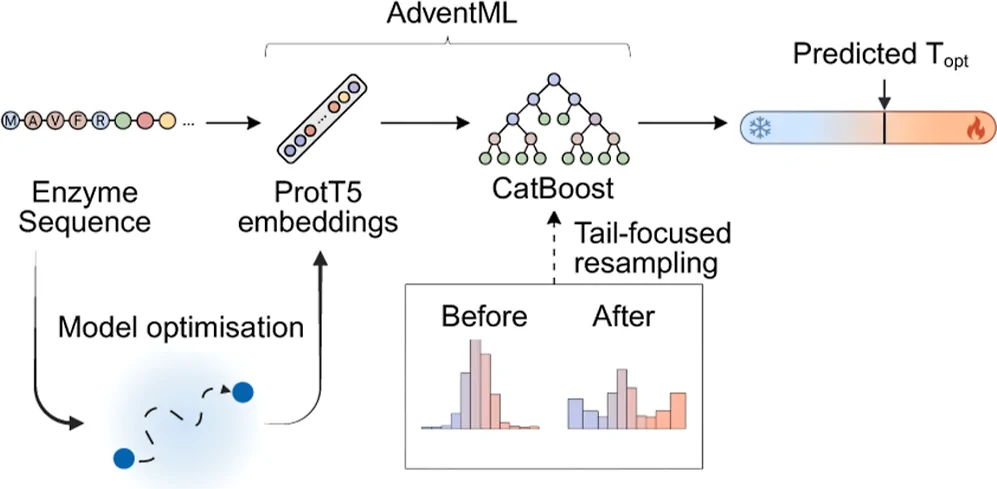

## 1 Introduction

Enzymes that are stable and active at high temperatures, are widely used in pharmaceuticals, food processing, chemical manufacturing,^1–8^ molecular biology and protein engineering.^9^ However, experimental determination of an enzyme’s optimal catalytic temperature (*T*_opt_) is costly, labor intensive, and time-consuming, highlighting the need for computational prediction methods.

Organism growth temperature (OGT) is often used to estimate protein thermostability and, by extension, the enzyme’s optimal catalytic temperature (*T*_opt_).^9,10^ However, OGT presents significant limitations for predicting enzyme performance at specific temperatures. First, OGT shows only a limited correlation of 0.48 to *T*_opt_ for single enzymes.^11^ Second, protein thermostability does not always reflect catalytic activity at high temperatures.^9^ Third, OGT data is not only lacking for many organisms but is also experimentally expensive to determine^9,12^ and is intractable for unculturable organisms. Moreover, many enzymes exhibit *T*_opt_ values substantially higher or lower than their organism’s OGT, making OGT merely a rough estimate. Both the need for better temperature estimates than OGT, and the cost and time related to experimental OGT or *T*_opt_ determination underline the need for accurate enzyme temperature prediction models.^13^

Thermal adaptation is primarily reflected at the protein level^14^ for unicellular organisms (animals have physiological adaptations). Numerous genomic and proteomic features correlate with OGT or *T*_opt_, including dinucleotide presence/absence,^15^ GC content in structural RNAs,^16^ amino acid composition of the proteome,^14,17^ IVYWREL correlation with OGT for Archaea and Bacteria,^9,17^ protein length,^18^ amino acid properties,^17,19,20^ and structural features.^21–23^ However, these factors are individually weak determinants of protein unfolding^24,25^ and show even smaller effects on catalytic temperature.^9^ Nevertheless, both the link between proteins and temperature, and existing temperature prediction models suggest that building accurate prediction models for *T*_opt_ based on protein characteristics is feasible.

Indeed, recent developments include *T*_opt_ prediction models both in the presence and absence of OGT, showing promising results. Nevertheless, existing approaches remain limited. Models like TOME and TOMER both require OGT data for *T*_opt_ prediction, severely limiting their applications.^9,10^ Additionally, some recent approaches targets specific enzyme classes, such as Preoptem.^26^ Recent improvements in *T*_opt_ prediction include work by Zhang et al. developing a Segment Transformer model, and Seq2Topt by Qiu et al. using ESM2-8M or Progen2-small with fine-tuning.^26,27^ However, prediction at temperature extremes remains challenging, and recent approaches increasingly use deep learning architectures, often without establishing simple baselines. Critical questions remain regarding model generalizability and out-of-distribution performance, particularly the accuracy at extreme temperatures, that are after all of greatest interest.

In this work, we address these challenges by systematically examining *T*_opt_ prediction across multiple dimensions: protein sequence representations, model complexity, data resampling strategies, and training–test similarity (Figure 1). We trained and compared both classical machine learning methods with deep learning based approaches. Surprisingly, we show that a mix of advanced and classical methods is optimal. We demonstrate that accurate *T*_opt_ prediction in the absence of OGT is achievable with recent protein language models (PLMs), while classical physicochemical properties alone prove insufficient. Although resampling methods showed mixed results overall, they provide some improvements at temperature extremes. To facilitate *T*_opt_ prediction for the research community, we released AdventML on GitHub.

**Figure 1:**
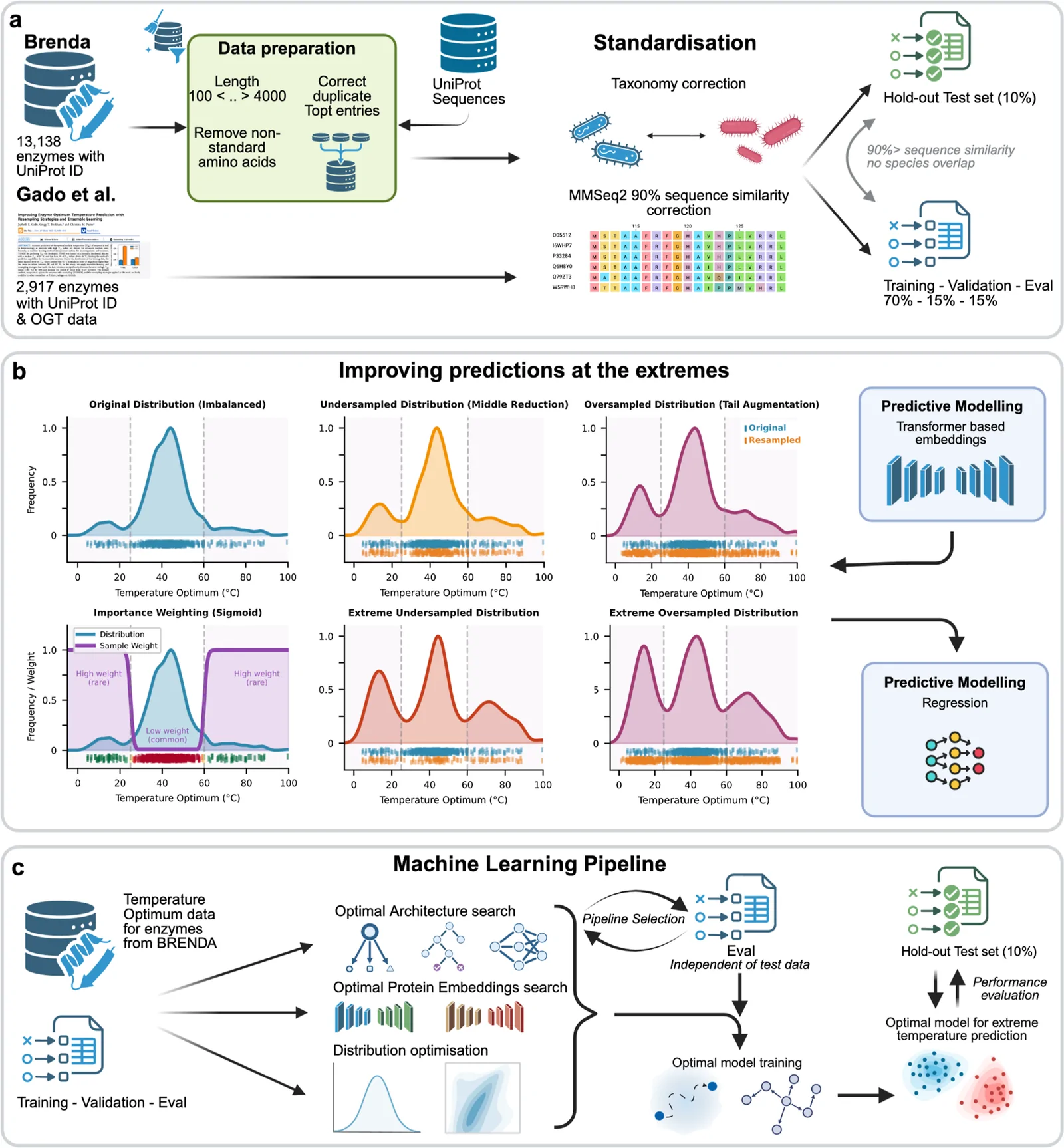
AdventML workflow for *T*_opt_ prediction. (a) Data collection and preprocessing from the BRENDA enzyme database, including quality filtering, deduplication, and taxonomic annotation. (b) Resampling strategies to improve predictions at temperature extremes. Both oversampling and undersampling approaches were evaluated, including extreme variants that target values <25 °C and >60 °C for oversampling, and >25 °C and <60 °C for undersampling, to balance training examples across the temperature range. (c) Systematic search for optimal machine learning strategy, including protein representation comparison, data balancing evaluation, and architecture comparison, with final assessment on an independent held-out test set with sequence and phylogeny similarity correction.

## 2 Results

The central questions in this research are whether *T*_opt_ prediction is possible in the absence of organism growth temperature (OGT) data and, if so, whether strategies tailored to extreme temperatures can improve the identification of thermostable and cold-adapted enzymes. To this end, we evaluate prediction performance with and without OGT across multiple protein representations, and we extend this comparison to the full training set of ∼9000 sequences. The data set spans broad taxonomic diversity and is concentrated in the mesophilic temperature range (Figure 2; Table S1). We further investigate extreme-regression strategies including resampling, bagging, and loss weighting to enhance performance in the cold and hot tails of the *T*_opt_ distribution. Finally, we compare several machine learning architectures, including deep learning, against classical machine learning.

**Figure 2:**
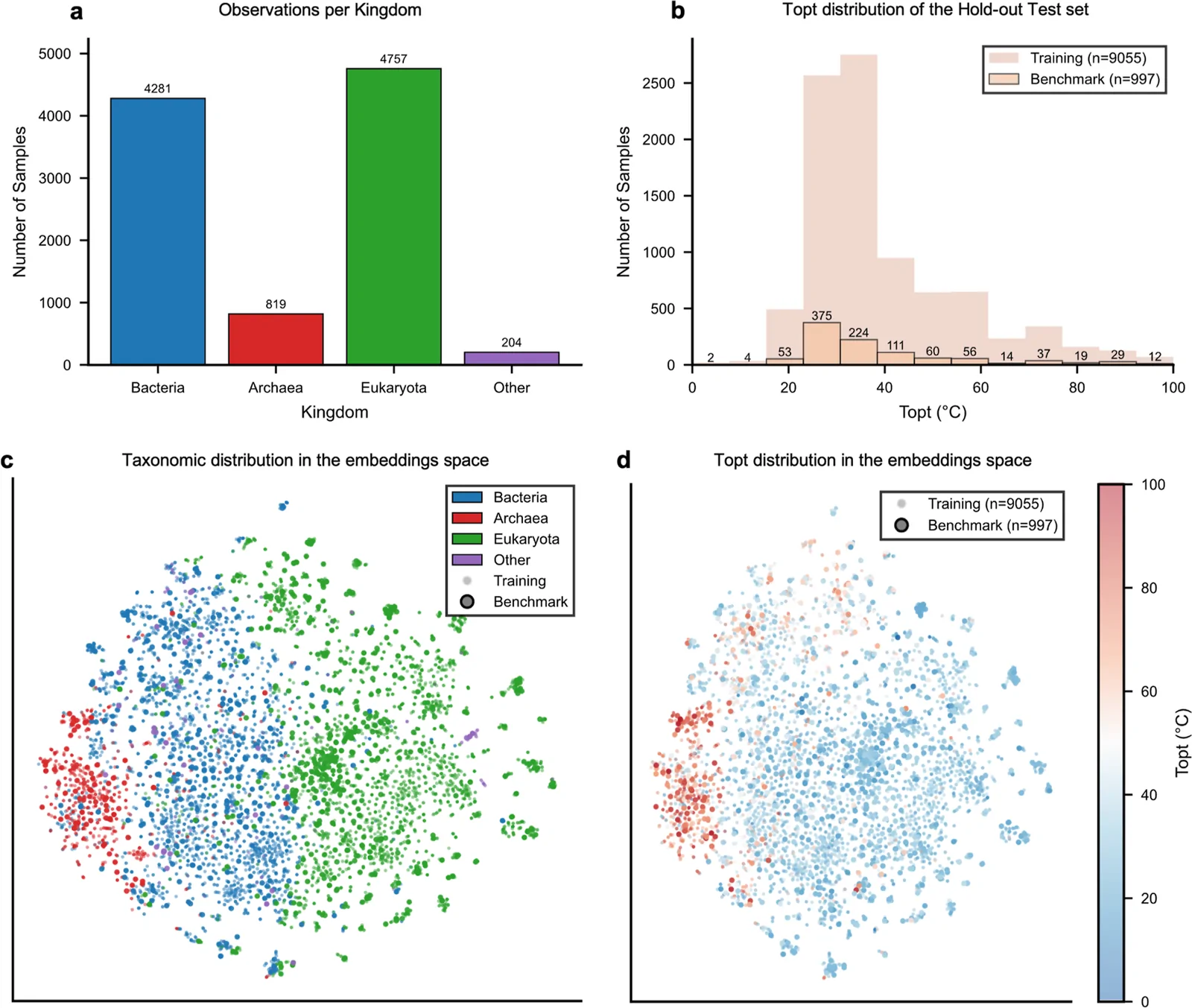
Phylogenetic and thermal diversity of the *T*_opt_ data set. (a) Taxonomic distribution of enzyme sequences across kingdoms in the filtered BRENDA data set. (b) Distribution of optimal temperature (*T*_opt_) values in the training and held-out test sets, showing concentration in the mesophilic range (30–50 °C) with sparse representation at temperature extremes. (c) t-SNE projection of ProtTrans-T5-XL-U50 embeddings colored by taxonomic kingdom, revealing distinct clustering by phylogenetic origin. (d) t-SNE projection of ProtTrans-T5-XL-U50 embeddings colored by *T*_opt_, demonstrating a smooth temperature gradient across the representation space that aligns with taxonomic structure. Descriptive statistics are available in Table S1.

### 2.1 Modest OGT Benefit for *T*_opt_ Prediction in State-of-the-Art PLMs

Many thermostability adapted enzymes have been discovered in, or originate from, temperature resistant organisms. In this context, the optimal growth temperature (OGT) can serve as a useful proxy for assessing enzyme thermostability. However, this relationship is not perfect: not all enzymes from thermophilic hosts are thermostable themselves, and conversely, thermostable enzymes can also arise from mesophilic (and even psychrophilic) organisms.

Here, we investigate the extent to which OGT contributes to predictive performance when coupled with modern protein representations derived from protein language models (PLMs) using the original TOMER training–test split.^10^ We evaluate both (i) traditional protein variables, such as physicochemical descriptors, and (ii) PLM based embeddings, using Histogram Gradient Boosting, CatBoost, and support vector regression (SVR) with hyperparameter tuning.

Overall, learned protein representations substantially outperform classical physicochemical descriptors in predictive performance (Figure 3). Notably, the performance gap between models trained with versus without OGT is small for state-of-the-art PLMs. The difference was smallest for ESMC, ESM3 and Pro-PRIME models. This difference, which is near zero for PRIME, was expected since this model is trained to represent and distinguish protein thermostability.^28^ Performance for top models, including ESM models and ProtTrans T5, was similar and hovered around *R* ^2^ ∼ 0.50–0.60 with an *R* ^2^ = 0.53 and MAE = 9.44 °C for ESM3 SM open and an *R* ^2^ = 0.52 with MAE = 9.60 °C for ProtT5-XL-50 in the absence of OGT (Table S2). Furthermore, the strongest relative improvement occurred for physicochemical features indicating that OGT can partially compensate for the limited sequence derived temperature signal in these protein representations.

**Figure 3:**
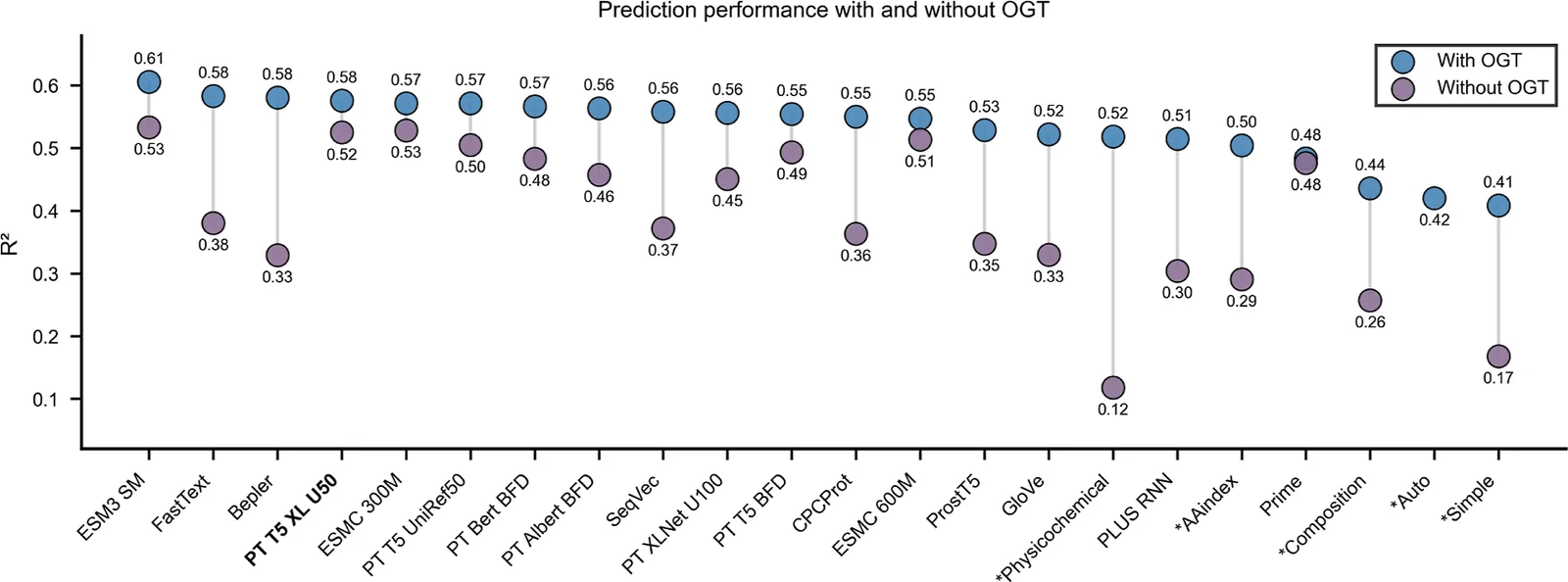
Impact of organism growth temperature on *T*_opt_ prediction across protein representations. Embeddings were computed for the TOMER data set (*n* = 2, 917 sequences). For each representation, Histogram Gradient Boosting, CatBoost, and support vector regression models were trained with hyperparameters optimized via Optuna, and performance was averaged across the three regressors. Modern protein language model embeddings show minimal performance gain from OGT inclusion, whereas classical physicochemical descriptors benefit substantially.

### 2.2 Marginal Differences between State-of-the-Art Protein Representations for *T*_opt_ Prediction

We benchmarked a broad set of protein representations to identify the most effective feature space for enzyme temperature prediction. In addition to the models described in Section 2.1, we expanded the panel of protein language models (PLMs) to include newer transformer based encoders, notably Ankh3 and ESM-2. Furthermore, models were evaluated across a diverse suite of regression models: Histogram Gradient Boosting, XGBoost, CatBoost, QuantileGBR, ElasticNet, support vector regression (SVR), and kernel ridge regression. All models were tuned and evaluated on the expanded BRENDA data set.

Across all regressors, transformer derived embeddings consistently delivered the strongest predictive performance (Figure 4). These PLM representations substantially outperformed classical physicochemical property descriptors and also improved performance over older learned representations based on convolutional neural networks (CNNs) or recurrent neural networks (RNNs). The best overall results were obtained with ProtT5-XL-U50, which achieved *R* ^2^ ≈ 0.53 with MAE = 7.93 °C (Table S3). While ProtT5-XL-U50 was the top performer, differences among the leading state-of-the-art protein representations were generally small, with most high performing PLMs achieving comparable performance. In contrast, physicochemical property based baselines performed substantially worse, underscoring the importance of current PLMs.

**Figure 4:**
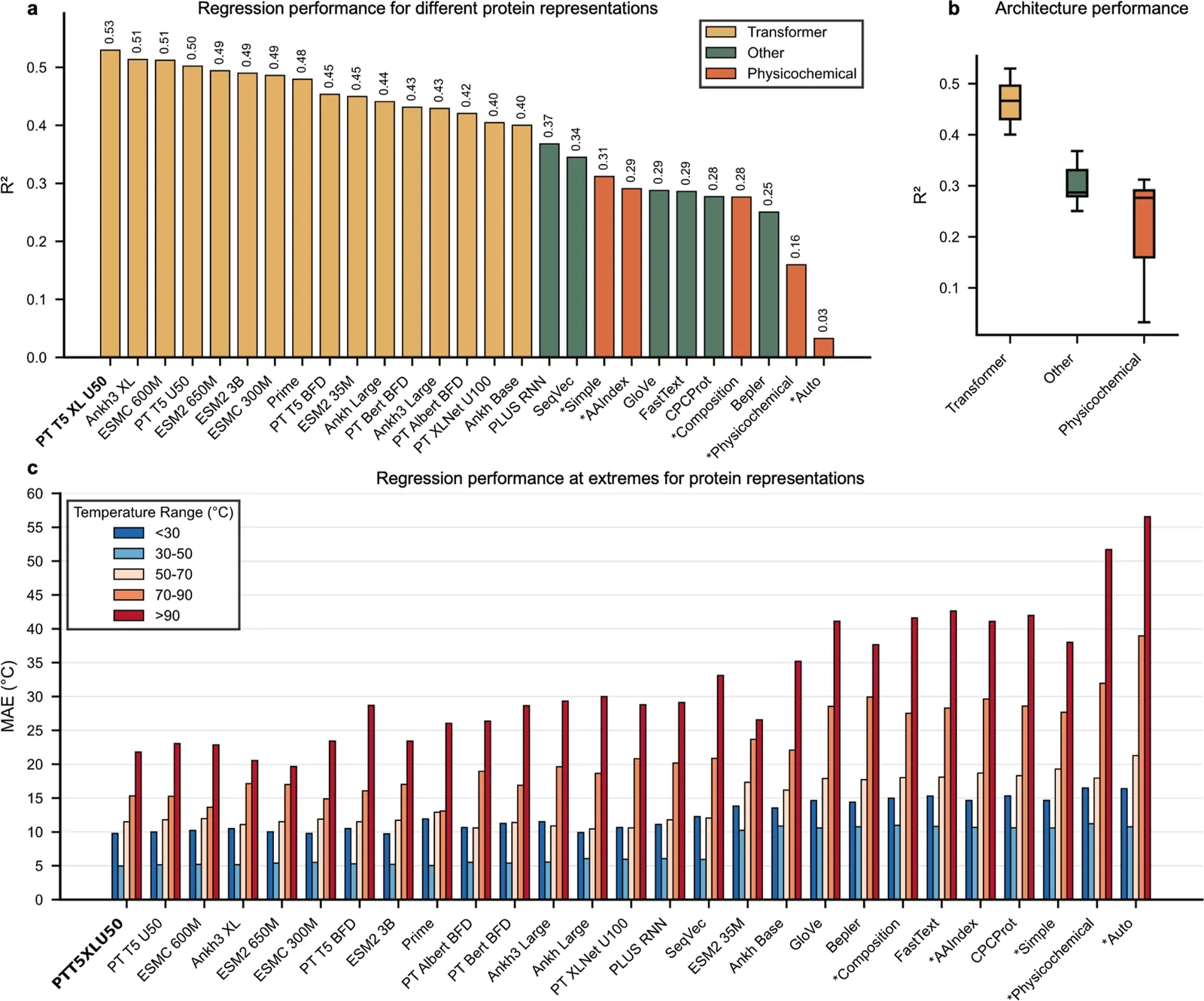
Protein representation comparison for *T*_opt_ prediction. (a) Coefficient of determination (*R* ^2^) across protein representations, with performance averaged over Histogram Gradient Boosting, XGBoost, CatBoost, QuantileGBR, ElasticNet, SVR and KernelRidge. All models were optimized with Optuna. (b) Performance grouped by representation architecture, comparing transformer-based protein language models (PLMs), other learned embeddings, and classical physicochemical descriptors. (c) Regression performance stratified by temperature range, showing that representation differences are most pronounced at temperature extremes.

Furthermore, differences in performance between protein representations are greater for extreme conditions (Figure 4c). Only PLM embeddings maintained acceptable prediction performance, whereas simpler physicochemical descriptors and older learned representations largely failed to identify enzymes adapted to extreme conditions (i.e. low predictive performance at extreme temperature ranges). Notice that PRIME, a domain adapted language model for temperature showed particularly strong performance in the 70–90 °C range, while ESM-2 based representations performed best for the most extreme temperature range (>90 °C), suggesting that different PLMs may capture complementary signals relevant to different temperature regions (Table S3). Additionally, performance did not show a clear trend between representation size and model accuracy (Table S4).

### 2.3 Resampling Improves Performance at Extremes with Minor Overall Prediction Degradation

To address the strong imbalance in the *T*_opt_ distribution and the resulting degradation of performance at the tails, we systematically evaluated a range of resampling strategies in combination with multiple regression models. Specifically, we benchmarked both over and under-sampling approaches using CatBoost, ElasticNet, Histogram Gradient Boosting, KernelRidge, QuantileGBR, support vector regression (SVR), and XGBoost.

We considered several classes of resampling implementations, including regression adapted algorithms such as SMOTER and WERCS, as well as “extreme” variants designed to focus explicitly on the tails of the temperature distribution. In these extreme protocols, undersampling was restricted to the central range (25–60 °C) to create a more balanced training set by reducing the prevalence of mesophilic observations. Conversely, oversampling was applied only to the extremes (<25 °C and >60 °C) to generate additional tail examples. For all resampling methods hyperparameters were tuned, while keeping the downstream learning algorithms fixed at sensible hyperparameter settings to limit overall search complexity.

Across models, the strongest resampling methods yielded broadly similar outcomes: error at the extremes decreased, while MAE increased slightly for values near the median (Figure 5). Notably, random oversampling and random undersampling targeted to the extremes performed close to the best performing algorithms, including extreme IHT and extreme ENN (Figure 5). These latter methods are undersampling approaches that explicitly filter out observations that are difficult to predict, which may correspond to noisy or ambiguous data points. The proximity in performance suggests that, in this setting, much of the benefits comes from rebalancing exposure to tail examples rather than advanced observation selection.

**Figure 5:**
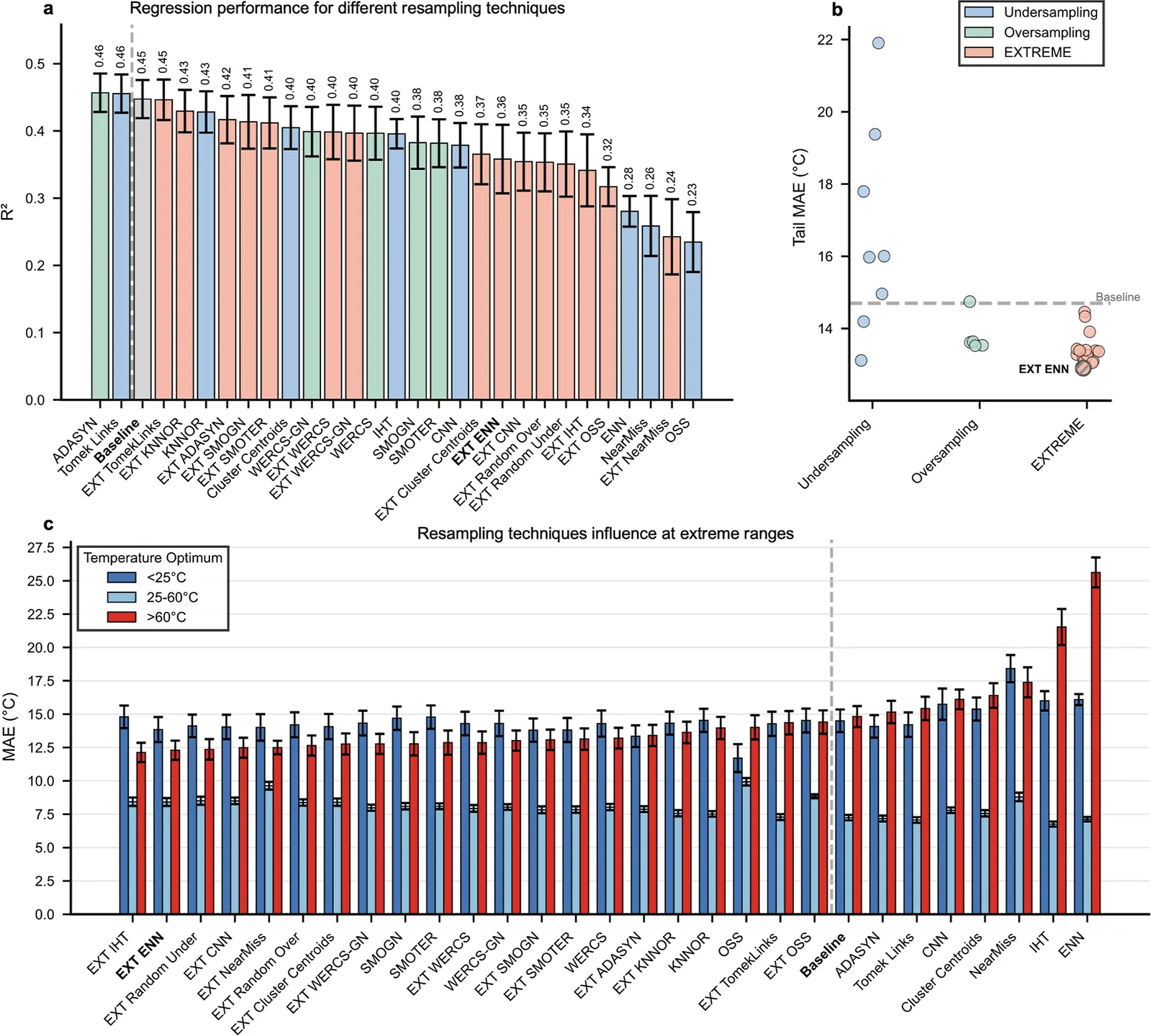
Resampling strategies for *T*_opt_ prediction. Models were trained using 5-fold cross-validation without sequence similarity correction. Resampling hyperparameters were optimized while keeping downstream regressors (Histogram Gradient Boosting, XGBoost, CatBoost, QuantileGBR, ElasticNet, SVR, and KernelRidge) at fixed settings. Extreme-focused implementations use masking to exclude values between 25 and 60 °C for oversampling and exclude values <25 °C and >60 °C for undersampling. (a) Overall *R* ^2^ comparison across resampling strategies on the held-out evaluation set. (b) Mean absolute error (MAE) difference relative to baseline, grouped by resampling strategy type (oversampling, undersampling, extreme-focused, and combined approaches). Tail MAE denotes MAE_<25 or >60_, calculated for observations below 25 °C or above 60 °C. (c) MAE stratified by temperature range.

Among oversampling techniques, SMOTER and SMOGN improved tail performance relative to the baseline, but they generally remained slightly behind the best undersampling approaches. Overall, differences between resampling algorithms were modest, and only limited improvement at extremes could be achieved, indicating that resampling alone cannot fully resolve the intrinsic difficulty of extrapolating to rare temperature ranges (Table S5).

Finally, we observed that the native IHT and ENN variants consistently worsened performance at the extremes. A plausible explanation is that, given the scarcity of extreme observations, these methods may treat tail points that lie close to mesophilic samples as hard to predict outliers and therefore remove them during instance selection. This behavior would further reduce already limited tail coverage and degrade the model’s ability to recognize enzymes adapted to extreme conditions.

Extreme-focused resampling produced clear tail gains but introduced a trade-off with global accuracy (Figure 5b). Extreme ENN achieved the strongest tail improvement (MAE_<25 or >60_ = 12.90 °C, Δ = −1.80 °C vs baseline) but reduced overall fit (*R* ^2^ = 0.358, MAE = 9.19 °C; Figure 5a). In contrast, extreme SMOTER and extreme WERCS provided supplementary extreme observations, reducing MAE_<25 or >60_ to ≈13.40–13.43 °C with smaller degradation in overall performance (Figure 5a,b).

### 2.4 Model Comparison

We next compared regression algorithms to determine which methods were most effective for temperature prediction at extremes (Figure 6). The highest evaluation *R* ^2^ values were achieved by GPR (0.560) and KernelRidge (0.559), followed by BaggingMLP (0.534) (Table S6). BaggingSVR and CatBoost showed comparatively strong performance above 90 °C, with MAE values of 13.19 and 13.28 °C, respectively.

**Figure 6:**
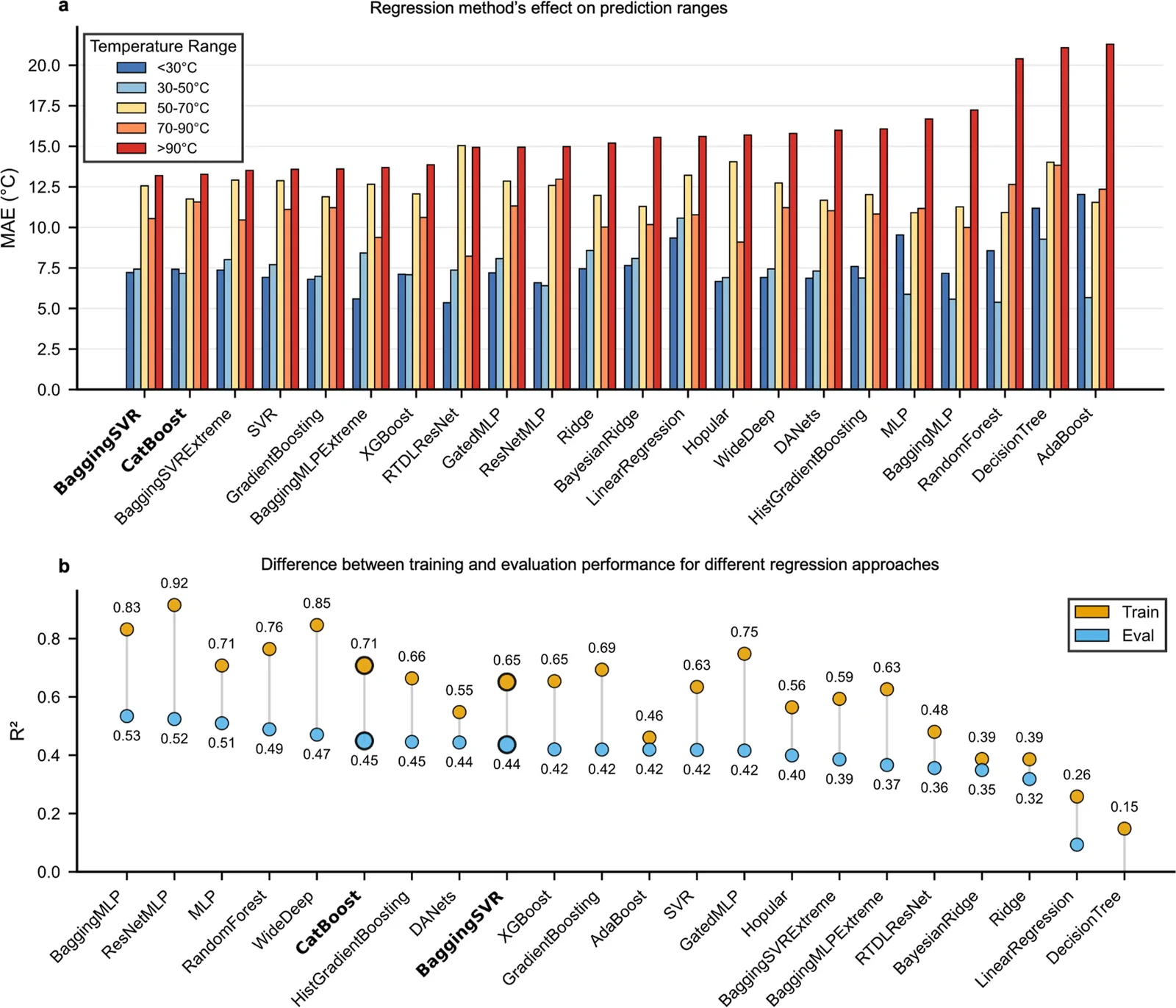
Regression model comparison for *T*_opt_ prediction. (a) Prediction performance across regression architectures stratified by temperature range, showing prediction error increases at temperature extremes for all models. (b) Difference between training and evaluation performance.

Notice that depending on the metric, many different architectures or models could be chosen. An example is GPR or KernelRidge, achieving *R* ^2^ ≈ 0.56 on the evaluation data (Table S6). However, even with regularization, it is clear that these models fail to capture trends given their large difference between training and evaluation performance. Furthermore, other models like ResNetMLP or MLP have both high *R* ^2^ values and relatively low gap between training and test performance, but fail to generalize at extreme temperatures. Improvements in global metrics did not consistently translate to improved predictions at extremes (Figure 6a).

Tail focused analyses showed clear trade-offs between global fit and predictions at extremes. Some methods (e.g., CatBoost) showed slightly worse overall MAE on the order of ∼1 °C relative to the best aggregate models, yet performed comparatively better at the extremes. Conversely, several ensemble and tree-based models that ranked highly by overall *R* ^2^ exhibited weaker performance at extremes, and showed worse generalization, even when measures were taken to mitigate overtraining.

Considering both overall accuracy, tail behavior, and generalization stability (as reflected by smaller train–evaluation differences), CatBoost emerged as a reliable choice for further exploration. Finally, prediction error increased with temperature range, with values >90 °C remaining the most challenging for all models (Figure 6a).

### 2.5 Comparison to Enzyme Temperature Prediction Models

Performance was benchmarked against the three state-of-the-art (SOTA) temperature prediction baselines using both the released models, and fully retrained models if possible (Figure 7a). For AdventML, top three major approaches were chosen for this final benchmark, training CatBoost with sigmoid importance weighting (AdventML), training CatBoost with sigmoid importance weighting and with extreme ENN undersampling (AdventML tail), and a final approach adding extreme WERCS oversampling in addition to extreme ENN (AdventML extreme).

**Figure 7:**
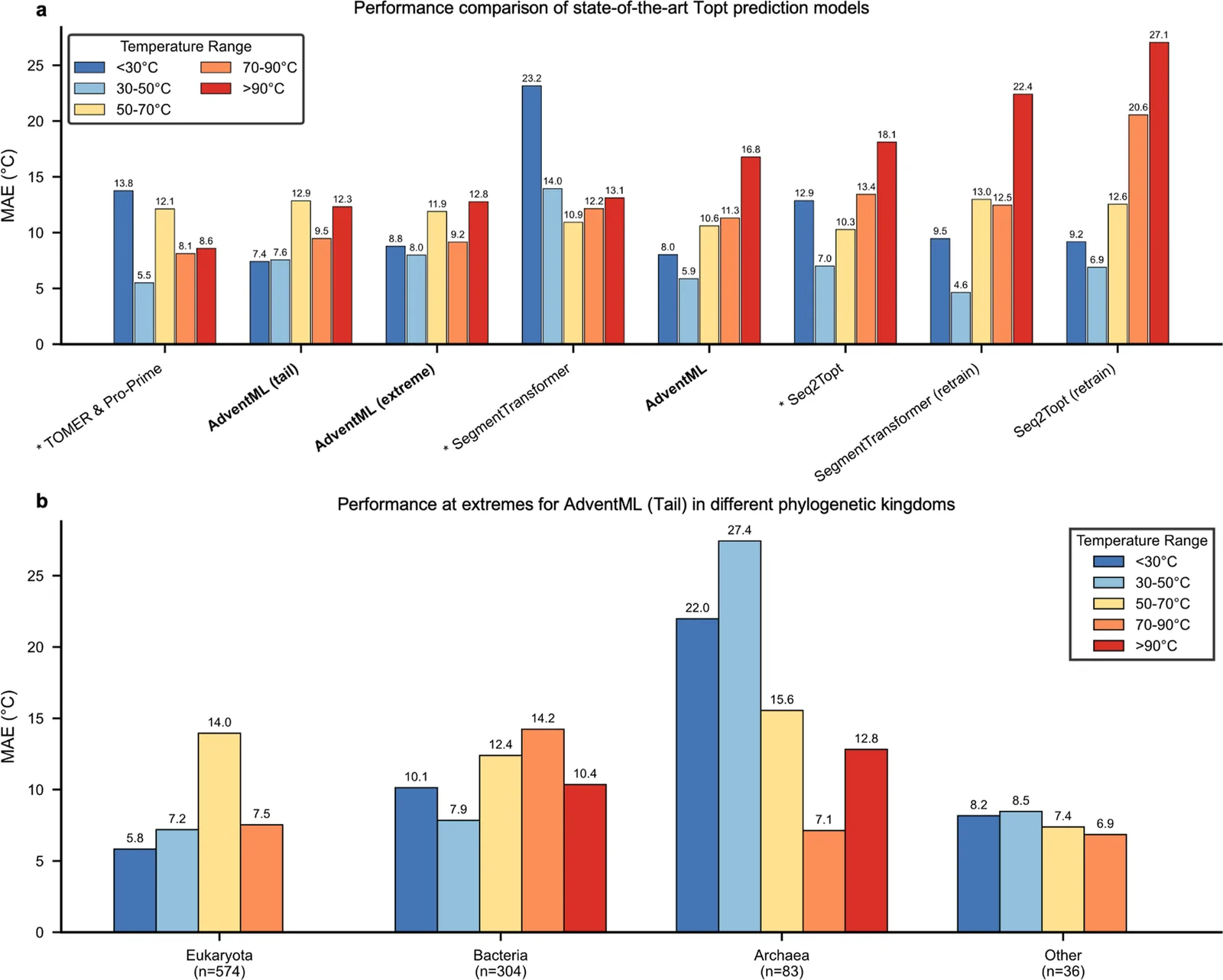
Comparison to state-of-the-art *T*_opt_ prediction models. (a) Performance comparison of AdventML variants against pretrained and retrained state-of-the-art models on the held-out test set. Pretrained models SegmentTransformer, TOMER and Seq2Topt may have seen part of the test data during original training, and are therefore not fully independent. AdventML models use CatBoost with Optuna hyperparameter optimization: the base variant uses no resampling, the tail variant applies extreme ENN undersampling, and the extreme variant combines extreme ENN with extreme WERCS for joint undersampling and oversampling. * indicates the models that have seen test data during training. (b) AdventML tail variant performance stratified by taxonomic kingdom. Limited observations in the held-out test set prevented evaluation of extreme temperature enzymes for Eukaryota.

Overall best performance was achieved by AdventML with *R* ^2^ = 0.646 and MAE = 7.58 °C (Figure 8). However, using a more balanced metrics and taking into account prediction at extremes (Figure 7) top performing models are AdventML tail (*R* ^2^ = 0.558, MAE = 8.45 °C), AdventML extreme (*R* ^2^ = 0.536, MAE = 8.85 °C) and the state-of-the-art combination of TOMER & Pro-Prime (*R* ^2^ = 0.605, MAE = 8.28 °C). TOMER with Pro-Prime slightly outperforms both AdventML tail and extreme at identifying temperature resistant enzymes (MAE_70–90_ = 8.13 °C vs 9.48 and 9.16 °C, respectively), but fails to accurately predict enzymes at lower temperatures (MAE_<30_ = 13.75 °C vs 7.42 °C for AdventML tail). Residual patterns for AdventML tail and extreme are shown in Figure S1.

**Figure 8:**
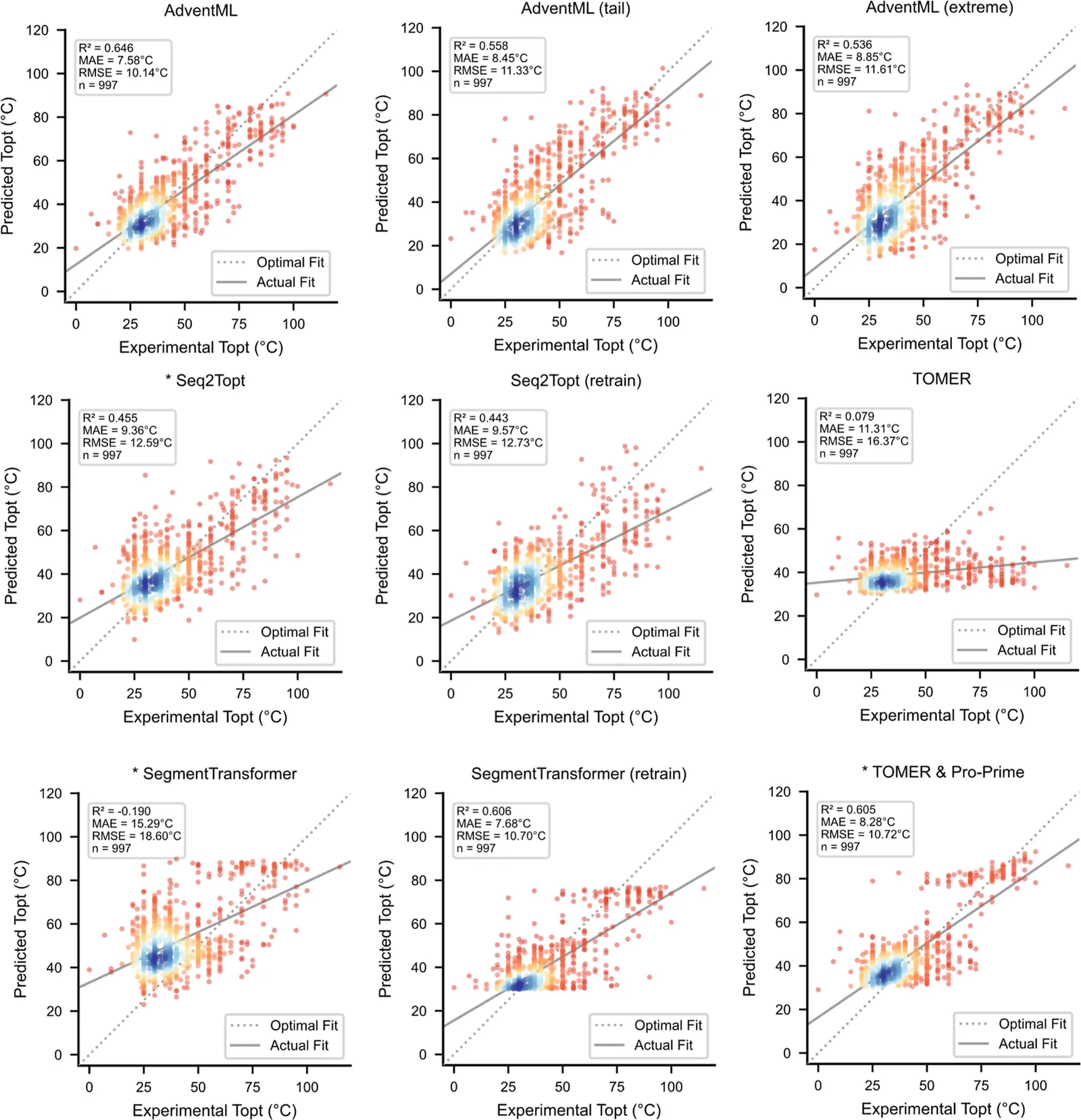
Predicted vs observed *T*_opt_ across all models. Scatter plot comparison of AdventML variants and state-of-the-art models on the held-out test set. Each panel shows predicted against observed *T*_opt_ values, with the diagonal representing perfect prediction. Points are colored according to their local density in observed–predicted plot, with blue indicating high-density regions and red indicating low-density regions. AdventML models use CatBoost with Optuna hyperparameter optimization: the base variant uses no resampling, the tail variant applies extreme ENN undersampling, and the extreme variant combines extreme ENN with extreme WERCS for joint undersampling and oversampling. The asterisk (*) indicates pretrained state-of-the-art models that have seen part of the test data during original training.

Overall, all AdventML model approaches outperform the state-of-the-art models Segment Transformer (*R* ^2^ = 0.606, MAE = 7.68 °C, retrained) and Seq2Topt (*R* ^2^ = 0.455, MAE = 9.36 °C). Surprisingly, only the TOMER & Pro-Prime combination outperformed our approach slightly but significantly for enzymes in the 70–90 range (Table S7). These results were expected for all SOTA models, since they have seen part of the held-out test data as training input during development. This effect is largest for the TOMER Pro-Prime combination, as this approach combines PLM specific temperature embeddings trained on Brenda *T*_opt_ data (Pro-Prime), which are then used as the OGT metric in the TOMER model, effectively trained twice on parts of the held-out test set. Given the model releases on GitHub, it was not possible to retrain every model from scratch to have a completely fair comparison without any overlap between SOTA training data and our held-out test set. Both the retrained Segment Transformer, TOMER, and TOMER & Pro-Prime combination showed a bias to overpredict temperatures.

Notice the shift in performance between original model weights for SOTA models, and the retrained variants (Figure 7). Almost all retrained SOTA model performed worse than its original model, an expected shift due to the complete independence of training and the held-out test set, while the original models had parts of the held-out test set as training input, leading to higher performance. For Seq2Topt, retraining shifted *R* ^2^ from 0.455 to 0.443 and MAE_>90_ degraded sharply from 18.12 to 27.05 °C. For Segment Transformer, the original model (*R* ^2^ = −0.190) improved dramatically to *R* ^2^ = 0.606 after retraining, indicating the released weights were not suited to this test distribution. TOMER without OGT performed poorly (*R* ^2^ = 0.079, MAE = 11.31 °C), confirming strong OGT dependence.

Differences between AdventML tail and extreme were significantly better on high temperatures than retrained SOTA models, while performance of the original Segment Transformer remained on par (Table S8). For temperatures above 90 °C (*n* = 25) there was no significant difference between TOMER & Pro-Prime, AdventML tail/extreme, and Segment Transformer despite the large difference in Figure 7 (see also Table S9). This was, however, expected due to the small sample size at 90 °C. Furthermore, only the TOMER & Pro-Prime combination was significantly better than our AdventML tail approach MAE_>70_, but was significantly worse at predicting cold-adapted enzymes than AdventML tail (MAE_<30_ = 13.75 vs 7.42 °C; Table S10) and has already seen testing data twice during training. For the test *R* ^2^ the AdventML approach was significantly better than all other approaches (Table S11).

Further statistical analysis revealed systematic bias in several SOTA models. A paired Wilcoxon signed-rank test indicated significant overprediction for Seq2Topt (HL bias = +2.271 °C, *p*_Holm_ = 7.57 × 10^–10^), SegmentTransformer (+11.973 °C, *p*_Holm_ = 1.31 × 10^–94^), TOMER (+2.160 °C, *p*_Holm_ = 2.67 × 10^–6^), and TOMER & Pro-Prime (+4.025 °C, *p*_Holm_ = 3.51 × 10^–35^). No significant directional bias was detected for AdventML (+0.183 °C, *p*_Holm_ = 1.000), AdventML tail (−0.633 °C, *p*_Holm_ = 0.269), AdventML extreme (−0.074 °C, *p*_Holm_ = 1.000), or for the retrained Seq2Topt and SegmentTransformer models (Table S12). Paired Cramér–von Mises testing detected significant overall distributional differences for every model (*p*_Holm_ = 9.0 × 10^–5^), although AdventML tail (CvM = 2.869) and AdventML extreme (CvM = 2.920) showed the closest overall agreement with the experimental distribution. Such differences are expected because regression tends to shrink extreme predictions toward the mean, producing narrower distributions despite corrections like resampling or weighted training (Figure S1). The largest local distribution gaps generally occurred around 30 °C: AdventML extreme had the smallest gap (*D* = 0.143), followed by AdventML (*D* = 0.168) and AdventML tail (*D* = 0.182), whereas SegmentTransformer had the largest gap (*D* = 0.588 at 37.5 °C). Overall, AdventML tail and extreme demonstrated closer distributional agreement than the SOTA models (Figure S2).

Residual distributions for all nine models are shown in Figure S3.

The Taylor diagram of the same nine models (Figure S4) summarizes global prediction performance as correlation, normalized standard deviation, and centered RMSE, with mean bias shown. AdventML lies closest to the observations among the low-bias models. AdventML tail and extreme recover observed variability (σ̂/σ≈1) at a modest cost in correlation and centered RMSE, consistent with tail-focused resampling. Pretrained SegmentTransformer and TOMER remain far from the observation; Seq2Topt, SegmentTransformer, and TOMER & Pro-Prime show the same overprediction also detected by the Wilcoxon test. Notice that the Taylor diagram is computed over all test proteins, it is dominated by the abundant mesophilic observations and does not indicate performance at extreme temperatures.

The additional metrics illustrate complementary aspects of predictive performance (Table S13). AdventML achieved the highest NSE, equivalent here to *R* ^2^ (0.646), and the lowest RMSE (10.14 °C). Its NSE corresponds to a 64.6% reduction in squared error relative to predicting the observed test-set mean for every protein. AdventML tail achieved the highest KGE (0.784 versus 0.755 for AdventML), reflecting closer reproduction of the observed temperature variability despite a slightly lower correlation (Figure S5). Collectively, the three AdventML variants achieved the best point estimates for NSE, RMSE, KGE and absolute PBIAS among the evaluated models, with complementary strengths in overall accuracy, reproduction of variability and aggregate bias.

### 2.6 Performance in Relation to Sequence and Phylogenetic Similarity

The data set spans broad phylogenetic diversity (Figure 2a), and low-dimensional structure reveals clear taxonomic organization: samples cluster by kingdom in protein feature space (Figure 2c), while *T*_opt_ exhibits a smooth gradient across this manifold (Figure 2d). These patterns suggest that both phylogeny and sampling density may influence generalization.

To quantify this effect directly, model performance stratified by phylogeny and by train–test similarity have been compared (Figures S6 and S7). Based on the simple metric of the lowest common ancestor (branch counting; range 1–14 ranks, mean = 3.1), no clear trend was observed in any model. Pearson correlations between phylogenetic distance and residuals were negligible for all AdventML variants (|*r*| ≤ 0.064) and all retrained SOTA models (|*r*| ≤ 0.055). The largest correlations occurred for TOMER (*r* = +0.180), Segment Transformer (*r* = +0.093) and Seq2Topt (*r* = +0.066), but they still showed barely any correlation. Furthermore, no deviation could be observed for models based on sequence similarity. Higher sequence similarity did not result in better predictions or a clear trend, with Pearson correlations between similarity and absolute prediction error near zero for all models (|*r*| ≤ 0.068). Only deviations can be observed for Segment Transformer with a tendency to predict temperatures higher than the actual experimental values (MBE = +11.75 °C), and a similar trend in the TOMER & Pro-Prime combination, but to a lesser degree (MBE = +3.72 °C).

## 3 Discussion

This work investigated whether enzyme optimal temperature prediction can be improved by combining protein language model (PLM) embeddings with resampling strategies and machine learning architecture search. The results demonstrate that PLMs outperform classical biology based features. Furthermore, resampling improved performance at extremes, with simple approaches performing better or on par with more complex algorithms. Finally, our best model combined CatBoost with extreme ENN undersampling to improve predictions for *T*_opt_ significantly compared to SOTA models.

The results demonstrate that PLM embeddings substantially outperform classical sequence-derived features for *T*_opt_ prediction. However, among the top-performing PLMs, differences were small, and neither model size nor publication date showed a consistent trend. For example, ESM-2 650M and 3B performed comparably, and newer models such as ESMC 300M did not outperform older models such as ProtT5 XL U50. This indicates that for state-of-the-art embeddings, practical considerations such as licensing and inference speed may be used as additional selection criteria, rather than parameter count or the most recent model (Table S4). A similar observation was made in literature, were smaller older models can outperform recent ones, especially on problems with limited data.^29,30^ Incorporating OGT as an auxiliary feature provided clear gains for classical representations, but only marginal improvements when combined with PLMs, indicating that PLMs already capture much of the organism level thermal information.

Other studies identified similar effects, with PLMs capturing organism level thermal information while classical representations do not.^31^ Furthermore, some studies indicated that the classical feature-based representations especially fail on thermophiles, while PLMs did not.^31,32^ Our results also showed no direct trend of increased performance with larger PLMs, which is in line with previous research showing midsized PLMs in combination with mean pooling outperforming other more complex approaches.^30^

An important notice is that while our results showed no clear benefit for very large PLMs, inevitably, predictions for the larger protein representations can become tricky due to the increased dimensionality. The larger PLMs like ProtTrans ALBERT BFD or Ankh3 XL had more than 2500 features, for only 10,000 observations. This inevitably increases overfitting difficulties, and size or appropriate protein representations will largely depend on number of observations, with larger protein representation better fitted to problems such as protein function prediction with many training observations.

Resampling consistently improved prediction at temperature extremes at the cost of a minor overall *R* ^2^ penalty, in line with findings reported for TOMER.^10^ The present study extended this analysis to a broader set of undersampling and hybrid over/undersampling methods, including WERCS and SMOGN. Even after hyperparameter optimization, targeted random undersampling in specific temperature ranges matched or outperformed these more complex approaches, indicating that complex resampling algorithms do not necessarily translate to prediction gains. Notably, resampling provided improvements beyond corrections such as weighted loss functions, underscoring its importance for rare event prediction.

The importance of resampling cannot be underestimated, with drastically worse predictions at extremes if absent. A similar trend can be seen in literature, showing that without corrections, rare continuous values will be underfit unless the distribution is rebalanced for machine learning training.^33^ Furthermore, studies have shown that rebalancing the distribution matter more than fancy interpolation.^34^ Our research showed a similar trend, with advanced interpolating (oversampling) methods like SMOGN or SMOTER underperforming compared to simple targeted random undersampling on extremes. The underlying reason is likely the challenge with oversampling: oversampling can introduce ambiguous observations and noise. Additionally, we identified also a trend were our adapted ENN undersampling approach improved over baseline prediction and targeted undersampling, but the default ENN implementation failed. ENN is an approach in which an observation is removed that disagrees the most with its neighbors. Applying this approach overall removes extremes since these are rare and can disagree with other mesophilic observations in the neighborhood. Adapting such algorithm to focus on only mesophilic observations rebalances the distribution, even better than random undersampling since it will not only remove mesophilic observations, but especially those that disagree with neighboring thermophilic or psychophilic observations. The extreme ENN approach was chosen as the final approach since it showed best improvements at extremes. For the AdventML extreme version, the combination of extreme ENN and WERCS was chosen to combine the best undersampling and oversampling approach to correct distribution imbalance even more.

Across machine learning types, large overall performance differences were observed, but variation among top-performing methods was minor. The final model, ProtT5 XL U50 combined with CatBoost and extreme ENN undersampling, achieved performance exceeding current state-of-the-art models. CatBoost was selected over BaggingSVR despite negligible performance differences, given its lower computational cost and greater interpretability. Advanced deep learning architectures such as gated MLP and ResNetMLP did not improve over gradient-boosted or kernel-based methods, likely due to the low observation-to-feature ratio limiting their generalization capacity. Furthermore, analyses stratified by phylogenetic distance and sequence similarity revealed no clear trend of prediction bias toward closer evolutionary relatedness, suggesting that the model generalizes robustly across the taxonomic range present in the data.

Current prediction performance appears to plateau around 0.5–0.6 *R* ^2^, and it remains unclear whether substantial further gains are achievable given inherent limitations in the underlying data. *T*_opt_ values, even when sourced from a single database such as BRENDA, are notoriously noisy: duplicate entries for the same enzyme frequently report widely different values, and key experimental variables such as buffer composition, substrate identity, pH, and assay or experimental duration, are often absent for predictions. As a result, predicting *T*_opt_ from protein sequence or structure alone is inherently an approximation of a context-dependent target variable. This challenge is further increased by the nature of thermal activity profiles, where enzymes can lack a sharp optimum and instead exhibit broad activity plateaus across a range of temperatures.^35^ Such profiles introduce substantial variability in both training labels and evaluation targets, effectively imposing a sort of ceiling on achievable prediction accuracy that reflects experimental noise rather than model deficiency.

This study has some important limitations. First, protein representations, resampling strategies and machine learning architectures were screened sequentially. A resampling method that works well with one representation or regressor may not be optimal with another.^34,36^ In particular, resampling hyperparameters were tuned while downstream regressors were held at fixed settings. Joint search of representation, resampling and architecture would address this, but at a large computational cost. Current search approach considered many architectural options, already at a cost of more than 20,000 CPU hours on Flemish Supercomputer Center, making such approach difficult. Second, the protein language models were used as frozen, mean-pooled embedding extractors. Such approach can be optimal for problems with limited training data,^30^ but inevitably loses interpretation as embeddings do not relate to specific sequence locations anymore. A single independent leakage-safe held-out test set was used to compare state-of-the-art models with AdventML models. This keeps evaluation independent, but makes it harder to estimate variability, repeated resampling of the test partition or Monte Carlo cross-validation could further quantify that uncertainty. Fourth, *T*_opt_ was modeled from sequence embeddings alone. Substrate, buffer, pH and assay conditions can shift the observed optimum by several degrees, yet paired metadata of this kind are rarely available in BRENDA, so an assay-conditioned model was not feasible here. Finally, data at temperature extremes remain sparse in both training and test data and are mostly bacterial. The >90 °C test subset is small, and eukaryotic extremes could not be evaluated. Tail metrics and cross-kingdom generalization should therefore be interpreted with caution. In addition, several released state-of-the-art models could not be fully retrained without overlap with our test set, which limits the fairness of those pretrained comparisons.

Several future research avenues could improve *T*_opt_ prediction. First, improved data curation is likely to yield the most direct gains. While the current work applied quality filtering, more extensive validation of training and test labels is warranted, particularly given recent reports of incorrect BRENDA *T*_opt_ annotations, including assay temperatures or pH recorded as optima.^37^ Automated literature extraction could both correct such entries and recover currently missing context (substrate, buffer, pH and assay duration).^38–40^ Second, related thermal properties available at larger scale than *T*_opt_, including melting temperature and organism growth temperature,^41,42^ could be used through transfer or multitask learning. DeepET showed that pretraining on OGT and fine-tuning on *T*_opt_ or *T*_m_ improves sequence-only thermal prediction.^43^ EpHod used a similar strategy for pH, pretraining on environmental pH before fine-tuning on catalytic pH_opt_.^44^ The same logic could be applied to *T*_opt_, with the caveat that *T*_opt_ and thermostability are only weakly correlated and can be uncoupled.^45,46^ Third, architectural changes remain open. The present study used frozen PLM embeddings; parameter-efficient fine-tuning of the language model itself often improves downstream protein prediction, especially on small labeled sets.^47^ Because differences among top PLMs were small, concatenating or otherwise combining complementary embeddings is a more promising next test than replacing ProtT5 with a newer model of similar size. Structure-aware models that combine sequence embeddings with geometric graphs have already been used for optimal temperature, pH and salt^32^ and are a more direct complement to sequence-only AdventML than physics-based molecular dynamics. Finally, two design choices in this study could be revisited: joint rather than sequential optimization of representation, resampling and architecture,^34,36^ and moving from a single *T*_opt_ point to thermal activity ranges or full activity–temperature profiles, which better match how many enzymes actually behave.^35^ Experimental validation of predicted extremes will be needed before those predictions can be used with confidence.

## 4 Conclusion

AdventML is a prediction framework for enzyme optimal catalytic temperature that combines protein language model embeddings with resampling strategies and classical machine learning. The results demonstrate that modern PLM embeddings render organism growth temperature largely redundant for *T*_opt_ prediction, and that extreme-focused resampling improves tail predictions at minimal cost to overall accuracy. Notably, CatBoost with ProtT5 XL U50 embeddings and extreme ENN undersampling overall outperformed current state-of-the-art models at extremes, including deep learning approaches specifically designed for temperature prediction. AdventML is freely available as a GitHub tool to support the identification of thermostable and cold-adapted enzymes for biotechnological applications.

## 5 Methods

### 5.1 Workflow Overview

A pipeline was developed to predict the enzyme optimal temperature (*T*_opt_) from protein sequences by examining different prediction strategies.

First, *T*_opt_ and sequence data were extracted from the BRENDA enzyme database and preprocessed, including quality filtering, deduplication, and taxonomic annotation. To prevent information leakage, sequences were clustered by identity and separated into a training set and a held-out test set, with both sequence similarity and organism-level constraints.

Second, a systematic comparison was performed across multiple protein representations (transformer-based embeddings, other learned embeddings, and classical physicochemical descriptors), resampling strategies for addressing temperature distribution imbalance, and regression architectures. Model selection happened through a staged approach by first selecting the optimal representation, then evaluating the optimal resampling approach, and finally selecting the best performing machine learning architecture for *T*_opt_ prediction (Table 1).

**Table 1:** Fixed and Varied Settings across the Staged AdventML Evaluation Pipeline

stage	primary setting varied	fixed settings	evaluation protocol	selected setting
1. OGT benchmark	OGT included versus omitted across 22 representations	HistGradientBoosting, CatBoost, and SVR (results averaged over three regressors), variable ML hyperparameters, OGT-annotated subset, paired OGT/no-OGT runs use identical splits within each representation, no data set-level resampling, no cross-validation	hyperparameters selected by two-tailed weighted validation MAE, comparison reported on the held-out test partition. *n*: trainval = 2,333, test = 584	not included in pipeline selection
2. Representation benchmark	27 protein representations across 7 regressors	OGT absent, variable ML hyperparameters, no data set-level resampling, no cross-validation, results averaged over seven regressors	hyperparameters selected on validation, representation ranking on eval. 70/15/15 split of the 9055 training pool. *n*: train = 6338 (a few representations 6339), val = 1358, eval = 1359	selected ProtTrans T5 XL U50
3. Resampling benchmark	29 data set-level resampling methods	ProtTrans T5 XL U50 (1024 dimensions), OGT absent, seven fixed-hyperparameter regressors (results averaged), 5-fold cross-validation	cross-validation on trainval (*n* = 9055), ranking uses CV scores. No model-fit sample weighting	selected baseline, extreme ENN, and extreme ENN + WERCS
4. Architecture benchmark	49 realized models: 29 conventional and 20 deep-learning architectures	ProtTrans T5 XL U50, OGT absent, no general prefit data set resampling, variable ML hyperparameters, no cross-validation	weighted validation objective and two-tailed model-fit weighting, Optuna uses val, model ranking uses eval. Conventional and deep-learning runs share the same split: *n*: train = 6338, val = 1358, eval = 1359 (train + val + eval = 9055). Six weighted-bagging variants are separate internal mechanisms	selected CatBoost
5. AdventML training	three CatBoost models with EXTREME ENN and/or WERCS	OGT-free 1024-dimensional ProtTrans T5 XL U50 representation, variable ML hyperparameters, no cross-validation	internal tuning on trainval (*n* = 9055, train = 7696, val = 1359), evaluation on the held-out test set (*n* = 997)	evaluated AdventML (baseline), AdventML-tail (EXTREME_ENN), and AdventML-extreme (EXTREME_ENN_WERCS)

First, the contribution of organism growth temperature (OGT) was evaluated by comparing matched OGT-present and OGT-absent inputs across 22 protein representations and three regressors on the OGT-annotated data set. Second, 27 protein representations were compared across seven conventional regressors without data set-level resampling. Third, 29 data set-level resampling strategies were evaluated using this fixed representation and seven regressors with fixed hyperparameters in 5-fold cross-validation. Fourth, the regression architecture was varied while retaining the selected representation. This architecture benchmark did not include general prefit data set resampling. And an optimal temperature prediction pipeline configuration was selected, final models were trained, and performance was benchmarked against existing state-of-the-art *T*_opt_ prediction methods on the held-out test set, independent from earlier architectural comparisons. Hyperparameter selection and evaluation were performed independently within each stage using the corresponding validation or cross-validation protocol. The settings varied and held fixed, data partitions, evaluation protocols, and decisions carried forward at each stage are summarized in Table 1.

### 5.2 Data Collection and Preprocessing

The optimal temperature (*T*_opt_) data set was obtained from the BRENDA enzyme database^48^ through extraction from the BRENDA JSON file (version 2025_01) with release date May 30, 2025. For each entry, *T*_opt_ values were retrieved together with organism name, UniProt accession, and experimental comments. Next, Taxonomy IDs were assigned using the ETE3 NCBITaxa toolkit^49^ based on organism names. UniProt sequences were retrieved from UniProt^50^ via the UniProt REST API using accession IDs; obsolete accessions were remapped if possible. Entries without UniProt IDs from BRENDA were matched with the EC number and organism ID, and flagged accordingly.

The raw data set underwent quality control adapted from ref 9. Sequences with fewer than 50 amino acids were excluded. Entries where *T*_opt_ values were annotated with “assay at” in the BRENDA comments field were removed to ensure true optimal temperatures rather than arbitrary assay conditions. Duplicate entries sharing UniProt identifiers, identical *T*_opt_ values, and matching EC number prefixes were merged. For enzymes with multiple *T*_opt_ values, mean and standard deviation were calculated; outlier values deviating by more than σ + 10 °C from the mean were excluded before computing the average. Only true *T*_opt_ not originating from average calculations or without duplicates were considered for a held-out test set.

To ensure robust evaluation and prevent data leakage, sequences were partitioned into training and test sets using sequence similarity clustering with MMseqs2.^51^ Sequences were clustered at 90% identity with a bidirectional 80% coverage constraint to ensure only true full-length homologues were grouped together, meaning that test sequences shared less than 90% identity with any training sequence. Additionally, organism-level constraints were added such that no species appeared in both training and test sets. The held-out test set contained 10% of the original data set, with the remaining 90% used for training. The test set (held-out set) was only used in the final evaluation comparing state-of-the-art models with full pipeline implementations, other analysis such as protein representation comparison or resampling methods comparison were done with only the training set.

For OGT prediction experiments, we utilized the data set from ref 10, containing 2917 organism temperature pairs. The original split from Tomer et al. was used for training and testing. Furthermore, the data set was used only to make a comparison between predictions with and without OGT for different protein representations. All data set statistics are available in Tables S1 and S14.

Temperature and sequence-length distributions were summarized using the number of observations (*n*), mean, standard deviation (SD), median, quartiles (Q1 and Q3), minimum, maximum, and mode. For temperature, we also reported the interquartile range (IQR = Q3 – Q1). Skewness and excess kurtosis were reported to describe distribution shape. Temperature summaries also included the number and percentage of observations below 25 °C and above 60 °C.

### 5.3 Protein Features and Representations

Protein sequences were encoded using multiple protein representations to examine the optimal feature space for *T*_opt_ prediction. Three major protein representation groups were examined, including transformer-based embeddings, other learned protein representations and biology or physicochemical derived protein features. For transformer-based embeddings, average pooling was used to create fixed-length sequence-level embeddings. We examined ESM-2 (650M and 3B parameter variants),^52^ ESM-C (600M), ESM-1,^53^ PRIME, xTrimoPGLM, ProtTrans models (T5-XL-U50, T5-UniRef50, BERT, ALBERT, XLNet),^54^ and Ankh variants.^55^ Other learned protein representations we used include SeqVec,^56^ UniRep,^57^ PlusRNN,^58^ CPCProt,^59^ Bepler, FastText,^60^ GloVe,^61^ Word2Vec.^62^ Furthermore, we calculated physicochemical properties using protlearn, including amino acid composition, pseudoamino acid composition (PAAC), conjoint triad descriptors (CTD), autocorrelation descriptors, and AAindex-derived properties. These interpretable baselines captured basic compositional biases without learned representations. All feature representations are listed in Table S15. Additionally, all feature dimensionalities are listed in Table S4.

### 5.4 Protein Embeddings Comparison

Training data was split with MMseqs2 at 90% sequence identity into training, validation, and evaluation sets (70%/15%/15%). Each representation was evaluated across seven regression models: HistGradientBoosting, XGBoost, CatBoost, Quantile Gradient Boosting, ElasticNet, support vector regression, and kernel ridge regression. Hyperparameters were optimized using Optuna^63^ with 96 trials, minimizing a two-tailed sigmoid-weighted MAE with hard cutoffs at 25 and 60 °C to emphasize performance at temperature extremes. All hyperparameters were selected based on validation set performance. Models were evaluated on the held-out evaluation set, and average performance across all regressors was computed to provide a representation-level comparison independent of model choice.

For each protein representation, we recorded its number of features (*D*) and the ratio of features to training observations (*D*/*N*_train_), where *N*_train_ is the number of training observations. We also calculated the standard deviation of evaluation MAE across the seven regression models (σ_MAE,eval_). This measures how strongly performance depends on the choice of regression model, rather than variability between repeated training runs.

### 5.5 *T*_opt_ Prediction in the Presence or Absence of Organism Growth Temperature

The influence of OGT on *T*_opt_ prediction was assessed for different feature sets. Data with annotated sequence, *T*_opt_, and OGT values (derived from the TOMER data set) were used with a 70/15/15 split.^10^ Three regressors (HistGradientBoosting, CatBoost, support vector regression) were trained in two modes: with OGT included as a feature and without OGT. Hyperparameter optimization with 96 trials followed the same sigmoid-weighted MAE objective. For each representation, performance was averaged across the three models to isolate the contribution of OGT.

### 5.6 Resampling Strategies

Enzyme *T*_opt_ values exhibited imbalance, with heavy concentration in the mesophilic range (30–50 °C) and sparse representation at low (<25 °C) and high (>60 °C) extremes. To improve model sensitivity across the full temperature spectrum, a selection of resampling algorithms for continuous data was evaluated on the training set only.

#### 5.6.1 Undersampling Methods

Condensed nearest neighbors (CNN), cluster centroids, edited nearest neighbors (ENN), instance hardness threshold (IHT), NearMiss, one-sided selection (OSS), and Tomek links.

#### 5.6.2 Oversampling Methods

SMOTER (SMOTE for regression), SMOGN (SMOTE with Gaussian noise), ADASYN, *k*-NN oversampling (KNNOR), and WERCS (weighted relevance-based combination strategy).

#### 5.6.3 Extreme-Focused Variants

Tail-specific versions of the above methods (e.g., extreme ENN, extreme SMOTER) that selectively oversample the rare low and high temperature domains (<25 °C and >60 °C) while undersampling the dense midrange (25–60 °C).

For 5-fold cross validation, per fold, features were standardized, the resampling strategy was applied, and seven regressors were trained. The trained regressors were Histogram Gradient Boosting, XGBoost, CatBoost, QuantileGBR, ElasticNet, SVR and KernelRidge. For each resampling strategy, several resampling hyperparameters were tried with a grid-search approach, keeping regression hyperparameters fixed at a sensible setting. Performance per fold was averaged over the seven regression models. All resampling approaches are listed in Tables S16 and S17.

Pseudocode for the resampling procedures is provided in Algorithms 1–16 in the Supporting Information.

### 5.7 Model Comparison and Evaluation

To evaluate *T*_opt_ prediction performance on ProtTrans T5-XL-U50 embeddings, a set of classical regression and deep learning architectures was benchmarked.

The conventional regression comparison comprised 29 model configurations, including six weighted-bagging configurations (Table S6). The standalone models included the following families. **Linear models:** Ridge, ElasticNet, Bayesian ridge, Huber, linear regression, passive aggressive, quantile regression, orthogonal matching pursuit (OMP), automatic relevance determination (ARD), and partial least squares (PLS). **Tree-based models:** decision tree, random forest, extra trees, gradient boosting, HistGradientBoosting, and AdaBoost. **Gradient boosting libraries:** XGBoost and CatBoost. **Kernel-based models:** support vector regression (SVR), kernel ridge, and Gaussian process regression (GPR). **Neural:** multi-layer perceptron (MLP). Weighted-bagging ensembles used three base learners, MLP, SVR, and decision tree, each evaluated with moderate and extreme bootstrap sampling dynamic ranges of 10× and 1000×, respectively. These produced the six configurations reported in Table S6, BaggingMLP, BaggingSVR, BaggingDecisionTree, and their corresponding extreme variants.

Furthermore, 20 tabular deep learning architectures were evaluated: baseline MLP, RTDL ResNet, RealMLP, TabM, gated MLP, sparse MLP, TabNet, LassoNet, GANDALF, DANets, TabR, Hopular, FT-transformer, SAINT, NODE, ResNet MLP, wide & deep, TabNet-inspired, attention MLP, and SNN. All deep learning architecture names are listed in Table S18.

Models were evaluated on the training data with a 70%–15%–15% split for training, validation and evaluation data. Hyperparameters for all architectures were optimized using Bayesian optimization via Optuna. Classical regression models used 192 TPE trials, whereas deep learning architectures utilized 100 TPE trials. Deep learning training relied on a maximum of 500 epochs with early stopping (30 epochs patience) and learning-rate reduction on plateau (15 epochs patience). All hyperparameters are listed in Table S19.

To address the severe target imbalance, sample weights were applied during training and hyperparameter optimization using a two-tailed sigmoid function with exact cutoffs at 25 and 60 °C. This resulted in higher unit weight at temperature extremes while down weighting importance in the mesophilic core. The dynamic weight range applied was 1000× (minimum weight 0.001) for classical regression models. For deep learning architectures, this range was intentionally narrowed to 100× (minimum weight 0.01) to preserve gradient magnitudes and training stability across large parameter spaces.

Performance was quantified using standard regression metrics: coefficient of determination (*R* ^2^), root-mean-square error (RMSE), mean absolute error (MAE), mean absolute percentage error (MAPE), mean bias error (MBE), and Spearman and Pearson correlation coefficients. Metrics were reported for the overall test set, isolated tail regions (*T*_opt_ < 20 °C or >60 °C), and within stratified temperature bins (<30, 30–50, 50–70, 70–90, >90 °C).

### 5.8 Performance Metrics

Performance was evaluated from out-of-sample predictions. Metrics used during model development were calculated from validation or out-of-fold cross-validation predictions, whereas final generalization performance was calculated from held-out test predictions.

Because *T*_opt_ is strongly concentrated in the mesophilic range, aggregate scores are dominated by mesophilic observations and do not, by themselves, characterize performance at the cold and hot tails (Figure 2b). Metrics were therefore reported both for the complete evaluation set and within predefined temperature subsets, following recommendations for imbalanced continuous targets.^34,36^ The number of observations in each subset was reported alongside its corresponding metric.

Let *y*_*i*_ and ${ŷ}_{i}$ denote the observed and predicted *T*_opt_ of protein *i*, respectively, with *n* paired observations and residual (eq 1)­

1 $$e_{i}={ŷ}_{i}-y_{i}$$

Thus, positive residuals indicate overprediction and negative residuals indicate underprediction. The means of the observed and predicted values are denoted by *y̅* and $\overset{®}{\hat{y}}$ , respectively.

Absolute prediction error was summarized by mean absolute error (MAE) and root-mean-square error (RMSE, eq 2)­

2 $$MAE=\frac{1}{n}\sum_{i=1}^{n}\left|e_{i}\right|,,RMSE=\sqrt{\frac{1}{n}\sum_{i=1}^{n}e_{i}^{2}}$$

Both metrics retain units of °C. MAE was used as the primary ranking measure and for temperature-stratified comparisons, whereas RMSE gives greater weight to large residuals.^64^

Systematic error was measured using mean bias error (MBE, eq 3)­

3 $$MBE=\frac{1}{n}\sum_{i=1}^{n}e_{i}$$

and percent bias (eq 4)^65,66^

4 $$PBIAS=-100\frac{\sum_{i=1}^{n}e_{i}}{\sum_{i=1}^{n}y_{i}}$$

Under the residual convention used here, positive MBE indicates overprediction. PBIAS follows the sign convention used in the Supporting Tables: positive values indicate aggregate underprediction and negative values indicate overprediction. Mean absolute percentage error (MAPE, eq 5) was calculated as^65^

5 $$MAPE=\frac{100}{n_{\neq 0}}\sum_{i\in I_{\neq 0}}\frac{\left|e_{i}\right|}{\left|y_{i}\right|},,I_{\neq 0}=\left\{i:y_{i}\neq 0\right\}$$

where $n_{\neq 0}=\left|I_{\neq 0}\right|$ . For the held-out comparison, the single observation with zero experimental temperature was excluded, giving *n*_≠0_ = 996. MAPE is undefined for *y*_*i*_ = 0 and becomes unstable near 0 °C; it was therefore treated as a supplementary descriptive diagnostic and was not used to rank models.

Goodness of fit was quantified by

6 $$R^{2}=1-\frac{\sum_{i=1}^{n}e_{i}^{2}}{\sum_{i=1}^{n}{\left(y_{i}-\mathrm{y̅}\right)}^{2}}$$

With this definition, *R* ^2^ (eq 6) is identical to the Nash–Sutcliffe efficiency (NSE).^65–68^ It equals one for perfect predictions, zero when predictions have the same squared error as the observed mean, and can be negative when predictions perform worse than that reference. This quantity is distinct from the squared Pearson correlation coefficient *r* ^2^, which is not applied. Pearson correlation *r* was used to measure linear association, and Spearman’s ρ was used to assess preservation of rank order (eq 7)­

7 $$r=\frac{\sum_{i=1}^{n}\left(y_{i}-\mathrm{y̅}\right)\left({ŷ}_{i}-\overset{®}{\hat{y}}\right)}{\sqrt{\sum_{i=1}^{n}{\left(y_{i}-\mathrm{y̅}\right)}^{2}\sum_{i=1}^{n}{\left({ŷ}_{i}-\overset{®}{\hat{y}}\right)}^{2}}},,\rho =corr\left(rank\left(y\right),rank\left(ŷ\right)\right)$$

Here, corr denotes Pearson correlation, with average ranks assigned to ties for Spearman’s ρ.

Relative squared error^68^ and the RMSE-standard-deviation ratio (eq 8) were defined as

8 $$RSE=\frac{\sum_{i=1}^{n}e_{i}^{2}}{\sum_{i=1}^{n}{\left(y_{i}-\mathrm{y̅}\right)}^{2}}=1-R^{2},,RSR=\frac{RMSE}{s_{y}}=\sqrt{\frac{n-1}{n}RSE}$$

where *s*_*y*_ is the sample standard deviation (eq 9)­

9 $$s_{y}=\sqrt{\frac{1}{n-1}\sum_{i=1}^{n}{\left(y_{i}-\mathrm{y̅}\right)}^{2}}$$

The predicted sample standard deviation $s_{ŷ}$ is defined analogously.

For the Taylor diagrams, centered RMSE (eq 10) was calculated as

10 $$cRMSE=\sqrt{\frac{1}{n}\sum_{i=1}^{n}{\left[\left({ŷ}_{i}-\overset{®}{\hat{y}}\right)-\left(y_{i}-\mathrm{y̅}\right)\right]}^{2}}$$

with its normalized form ncRMSE = cRMSE/σ_*y*_, where $\sigma_{y}=\sqrt{\left(n-1\right)/n}s_{y}$ is the population-form standard deviation of the observed values. In a normalized Taylor diagram, the radial coordinate is $\alpha =s_{ŷ}/s_{y}$ , the angular coordinate is determined by Pearson *r*, and ncRMSE is represented by the distance from the observed reference point.^69^

Kling–Gupta efficiency (KGE, eq 11) was used to separate association, variability, and mean bias^65,66,70^

11 $$KGE=1-\sqrt{{\left(r-1\right)}^{2}+{\left(\alpha -1\right)}^{2}+{\left(\beta -1\right)}^{2}}$$

where

12 $$\alpha =\frac{s_{ŷ}}{s_{y}},,\beta =\frac{\overset{®}{\hat{y}}}{\mathrm{y̅}}$$

The optimum value is one. Because Celsius temperature is an interval scale with an arbitrary zero, β, PBIAS, MAPE, KGE, and the Theil coefficient defined below depend on the chosen temperature origin (0 °C).^71,72^ Errors near 0 °C are weighted more, and as such should be interpreted with caution.

Willmott’s refined index of agreement *d*_r_ (eq 13) was calculated as^70,73^

13 $$d_{r}=\{\begin{array}{ll} 1-\frac{\sum_{i=1}^{n}\left|e_{i}\right|}{2\sum_{i=1}^{n}\left|y_{i}-\mathrm{y̅}\right|} & \sum_{i=1}^{n}\left|e_{i}\right|\leq 2\sum_{i=1}^{n}\left|y_{i}-\mathrm{y̅}\right|\\ \frac{2\sum_{i=1}^{n}\left|y_{i}-\mathrm{y̅}\right|}{\sum_{i=1}^{n}\left|e_{i}\right|}-1 & otherwise \end{array}$$

The index lies in [−1, 1], with one denoting perfect agreement.

The Theil inequality coefficient was specifically calculated using the *U*_1_ definition (eq 14)^68^

14 $$U_{1}=\frac{RMSE}{\sqrt{\frac{1}{n}\sum_{i=1}^{n}{ŷ}_{i}^{2}}+\sqrt{\frac{1}{n}\sum_{i=1}^{n}y_{i}^{2}}}$$

Lower *U*_1_ indicates smaller error relative to the magnitudes of the two series.

Let *R*_*y*_ = *y*_max_ – *y*_min_. Range-normalized error and the overall index (eq 15) were defined as^68,74^

15 $${nRMSE}_{R}=\frac{RMSE}{R_{y}},,OI=\frac{1}{2}\left[2-{nRMSE}_{R}-RSE\right]=\frac{1}{2}\left[1+R^{2}-{nRMSE}_{R}\right]$$

Combined accuracy (eq 16) follows the implementation used for the reported tables

16 CA=0.33(RMSE+MAE+RSE)

Because RMSE and MAE are in °C while RSE is dimensionless, CA has no coherent physical unit and was treated as a descriptive composite only.

Higher *R* ^2^, NSE, KGE, *d*_r_, and OI indicate better agreement, whereas lower MAE, RMSE, MAPE, RSE, RSR, *U*_1_, |PBIAS|, and CA indicate smaller error. The ideal values of α and β (eq 12) are one, and the ideal MBE is zero.

ECDF plots were used to examine differences between the observed and predicted marginal distributions; these graphical diagnostics were not treated as additional scalar accuracy scores.

We reported the median prediction residual as an additional measure of bias. To examine how errors changed across the temperature range, observations were divided into ten equal-frequency bins based on experimental temperature. Within each bin, we plotted the median residual and the interval between the first and third quartiles (IQR). Positive residuals indicate overprediction.

MAE was further calculated in five nonoverlapping temperature bins (<30, 30–50, 50–70, 70–90, and >90 °C) and for combined tail subsets. Combined tail metrics use *T*_opt_ < 20 °C or *T*_opt_ > 60 °C for the feature-set and architecture benchmarks (conventional and deep learning), final predictive-model evaluation, prediction-versus-experiment comparison, and bootstrap tail metrics, denoted MAE_<20 or >60_ and $R_{<20\text{or}\;>60}^{2}$ . The resampling-strategy benchmark and its associated combined tail metrics instead use *T*_opt_ < 25 °C or *T*_opt_ > 60 °C, denoted MAE_<25 or >60_ and $R_{<25\text{or}\;>60}^{2}$ . Training resampling itself also defines tails as *T*_opt_ < 25 °C or *T*_opt_ > 60 °C, even though the resulting final models’ tail metrics use *T*_opt_ < 20 °C or *T*_opt_ > 60 °C. Exact end point inclusion followed the implemented bin definitions. Pairwise comparisons of model errors and residual-location tests are described in Sections 5.11 and 5.12, respectively.

The supplementary metrics were computed from the same *n* = 997 paired held-out predictions used in the state-of-the-art comparison. Scores are reported as point estimates with paired-protein percentile 95% confidence intervals (*B* = 10,000). Numerical values for all nine models are given in Tables S20 and S13.

### 5.9 AdventML Pipeline Evaluation

The model CatBoost was combined with the ProtTrans T5 XL UniRef50 embeddings and tested with extreme ENN and extreme ENN/WERCS for the development of the final *T*_opt_ prediction model. Models AdventML, AdventML tail and AdventML extreme were developed, using CatBoost without resampling, with extreme ENN resampling, and with the combination of extreme ENN and extreme WERCS resampling.

Hyperparameters were optimized using Optuna with 192 TPE trials, maximizing weighted *R* ^2^ on the validation set (15% of training data). Final models were retrained on the full resampled training data with optimized hyperparameters and sigmoid sample weighting (cutoffs 25/60 °C, dynamic range 100×), then evaluated on the held-out test set.

### 5.10 Comparison to State-of-the-Art

External baseline methods were retrained and evaluated on the same leakage-safe data splits to enable direct comparison. These included:

- **Seq2Topt**: ESM-2 encoder (esm2_t6_8M_UR50D, 8M parameters) with multihead attention and residual dense blocks, trained with MSE loss and Adam optimizer, using min–max target scaling to the 0–120 °C range.^75^
- **TOMER**: Rebagg ensemble of 100 decision tree regressors using amino acid composition (20 features) and OGT (1 feature) as input, with sigmoid-relevance resampling. Evaluated both with annotated OGT and with a default OGT of 37 °C when OGT was unavailable.^10^
- **SegmentTransformer**: multiscale segment attention network with a frozen ESM-2 (150M) backbone. Sequences truncated to 1000 residues.^26^
- **PRIME**: pretrained protein language model (AI4Protein/Prime_690M) used as an OGT predictor. Predicted OGT values were supplied as input features to TOMER when experimental OGT was unavailable.^28^

Each baseline produced test set predictions aligned with the AdventML evaluation framework, enabling head-to-head comparison under identical data partitions and evaluation metrics.

### 5.11 Statistical Significance Testing

Statistical significance of performance differences between models was assessed using the paired bootstrap resampling test following the methodology of Koehn et al.^76,77^ All state-of-the-art models were compared, including AdventML variants and retrained baselines. For each of the unique model pairs, *B* = 100,000 bootstrap replicates were drawn by sampling *N* = 997 aligned test predictions with replacement, using identical indices for both models in each replicate to preserve the paired structure. For each bootstrap sample, the corpus-level metric was computed for both models and the number of wins for each model were recorded. Tied scores for a given bootstrap sample were counted as 0.5 for both models. Two-sided *p*-values were computed as p=2×min(p̂,1−p̂) , with *p̂* the proportion of bootstrap replicates in which model A achieved a better score than model B. To control the false discovery rate across all pairwise comparisons for each metric, *p*-values were adjusted using the Benjamini–Hochberg procedure at α = 0.05.^78^ Comparisons were performed for *R* ^2^, MAE, and MAE within temperature subsets (<30 °C, >70 °C, >90 °C).

### 5.12 Paired Bias and Marginal-Homogeneity Tests

For each of the nine models, systematic prediction bias was assessed using the same *n* = 997 held-out protein pairs. Residuals were defined as $d_{i}={ŷ}_{i}-y_{i}$ , where ${ŷ}_{i}$ and *y*_*i*_ denote predicted and experimental optimum temperatures, respectively. Two-sided Wilcoxon signed-rank tests evaluated whether the residual distribution was symmetric about zero.^79^ Zero residuals were excluded, tied absolute residuals received average ranks, and *p*-values were calculated using a tie- and continuity-corrected normal approximation. Bias was summarized by the Hodges–Lehmann pseudomedian and its unadjusted 95% confidence interval, obtained using the asymptotic procedure in R’s wilcox.test; positive estimates indicate overprediction.

Equality of the experimental and predicted marginal distributions was assessed using paired Cramér–von Mises (CvM)^80^ and Kolmogorov–Smirnov (KS) tests. Let *F*_*n*_ and ${\mathrm{F̂}}_{n}$ denote the corresponding empirical cumulative distribution functions, $\Delta_{n}=F_{n}-{\mathrm{F̂}}_{n}$ , and $H_{n}=\left(F_{n}+{\mathrm{F̂}}_{n}\right)/2$ . The statistics were

$$T_{CvM}=n\int \Delta_{n}{\left(t\right)}^{2}\;dH_{n}\left(t\right),,D_{KS}=\sup_{t}\left|\Delta_{n}\left(t\right)\right|$$

Alongside the KS statistic, we reported a temperature at which the absolute difference between the observed and predicted empirical cumulative distributions was largest (*T*_D_)­

$$T_{D}\in \underset{t}{\mathrm{argmax}}\left|F_{n}\left(t\right)-{\mathrm{F̂}}_{n}\left(t\right)\right|$$

Null distributions were approximated using *B* = 100,000 centered Rademacher multiplier-bootstrap replicates (master seed 2024)

$$G_{b}^{*}\left(t\right)=\frac{1}{\sqrt{n}}\sum_{i=1}^{n}\xi_{ib}\left[1\left(y_{i}\leq t\right)-1\left({ŷ}_{i}\leq t\right)-\Delta_{n}\left(t\right)\right]$$

where the independent multipliers ξ_*i*b_ equal −1 or +1 with equal probability. Applying one multiplier to each protein’s paired contribution preserved within-pair dependence. Bootstrap statistics ∫*G*_b_^*^(*t*)^2^ d*H*_*n*_(*t*) and $\sup_{t}\left|G_{b}^{*}\left(t\right)\right|$ were compared with *T*_CvM_ and $\sqrt{n}D_{KS}$ , respectively. Monte Carlo *p*-values were calculated as (1 + *E*)/(*B* + 1), where *E* is the number of bootstrap statistics at least as large as the observed statistic. Holm’s step-down adjustment was applied separately across the nine models within each test family (Wilcoxon, CvM, and KS).

### 5.13 Sequence Similarity and Taxonomic Distance

We examined whether prediction errors were associated with similarity to the training data. For each test sequence, sequence similarity was summarized by its highest sequence identity to a training sequence. Taxonomic distance was measured using the number of taxonomic ranks to the lowest common ancestor with the closest training organism. Pearson correlation was used to assess the relationship between each distance or similarity measure and prediction error.

## Supplementary Material



## Data Availability

The AdventML prediction models and code are freely available on GitHub at https://github.com/LegallyOverworked/adventml. The processed *T*_opt_ data set and trained models, with their outputs, are available at 10.5281/zenodo.20842383.

## Notes

Additional benchmark tables for OGT comparison, protein representation comparisons, resampling strategies, model comparisons, pairwise significance tests, residual analyzes, sequence-similarity and phylogenetic-distance analyzes, protein representation descriptions, hyperparameter search spaces, and resampling method definitions/pseudocode (PDF)
