# Supplementary material for: AdventML: Advanced Enzyme Temperature Prediction with Transformer-Based Embeddings and Resampling Strategies
Source: J Chem Inf Model. 2026 Oct 12;66(20):13218–36. doi: 10.1021/acs.jcim.6c02109 (PMC13746777; doi:10.1021/acs.jcim.6c02109)
Supplement: Supplementary file 1 [file ci6c02109_si_001.pdf]

Supplementary Information

Table S1: Descriptive statistics for experimental T_{opt} and organism growth temperature (OGT). Temperature summaries are in °C. Q1-Q3 denotes the interquartile range endpoints; IQR is their difference. Tail columns report counts and percentages.

A. Location and spread

Dataset	Split	Variable	n	Mean	SD	Median	Q1-Q3	Min-Max
BRENDA	Train/ validation	T_{opt}	9055	40.2	15.7	37.0	30.0-45.0	0.0-120.0
BRENDA	Held-out test	T_{opt}	997	39.8	17.1	35.5	30.0-45.0	0.0-115.0
BRENDA	Total	T_{opt}	10052	40.1	15.9	37.0	30.0-45.0	0.0-120.0
TOMER	Training	T_{opt}	2333	44.7	18.5	37.0	30.0-55.0	0.0-120.0
TOMER	Test	T_{opt}	584	45.2	18.5	37.0	30.0-55.0	7.5-100.0
TOMER	Total	T_{opt}	2917	44.8	18.5	37.0	30.0-55.0	0.0-120.0
TOMER	Training	OGT	2333	41.0	20.4	33.0	28.0-44.0	4.0-99.0
TOMER	Test	OGT	584	40.5	18.7	34.0	28.0-42.2	9.0-96.0
TOMER	Total	OGT	2917	40.9	20.0	33.0	28.0-43.0	4.0-99.0

B. Distribution shape and temperature tails

Dataset	Split	Variable	IQR	Mode (n)	Skew	Excess kurtosis	$< 25\,°C$	$> 60\,°C$
BRENDA	Train/ validation	T_{opt}	15.0	37.0 (2057)	1.35	1.83	608 (6.7%)	949 (10.5%)
BRENDA	Held-out test	T_{opt}	15.0	30.0 (231)	1.53	2.02	62 (6.2%)	113 (11.3%)
BRENDA	Total	T_{opt}	15.0	37.0 (2199)	1.38	1.86	670 (6.7%)	1062 (10.6%)
TOMER	Training	T_{opt}	25.0	37.0 (437)	0.98	0.33	108 (4.6%)	412 (17.7%)
TOMER	Test	T_{opt}	25.0	37.0 (111)	0.95	0.09	28 (4.8%)	109 (18.7%)
TOMER	Total	T_{opt}	25.0	37.0 (548)	0.97	0.28	136 (4.7%)	521 (17.9%)
TOMER	Training	OGT	16.0	37.0 (283)	1.40	0.75	184 (7.9%)	403 (17.3%)
TOMER	Test	OGT	14.2	37.0 (73)	1.39	0.73	38 (6.5%)	96 (16.4%)
TOMER	Total	OGT	15.0	37.0 (356)	1.40	0.77	222 (7.6%)	499 (17.1%)

The BRENDA T_{opt} labels are mesophilic and right-skewed (train/validation: $n = 9055$, mean $40.2\,°C$, median $37.0\,°C$, Q1-Q3 30.0-$45.0\,°C$). Only 6.7% of training values are $< 25\,°C$ and 10.5% are $> 60\,°C$. The held-out test set ($n = 997$) matches this shape (mean $39.8\,°C$, same interquartile range), with a slightly larger SD (17.1 vs. $15.7\,°C$). Reported optima heap at $37\,°C$ in training and at $30\,°C$ in the test set. The TOMER set is warmer and more dispersed (mean $\approx 45\,°C$; $18\% > 60\,°C$), and OGT is systematically lower than T_{opt} (median 33 vs. $37\,°C$). Sequence lengths are those of typical globular enzymes (BRENDA median 413 aa, Q1-Q3 311-549 aa) with a long right tail; training and test length distributions agree.

Table S2: Effect of OGT features on prediction performance across feature sets. Evaluated on the original TOMER train-test split (2,917 samples). Performance values are averaged over HistGradient-Boosting, CatBoost, and SVR, each optimised with 96 Optuna trials. PLM embeddings were obtained via average pooling.

	**OGT**			**no OGT**			**|Δ OGT - no OGT|**		
Protein Representations	MAE	RMSE	R^2	MAE	RMSE	R^2	MAE	RMSE	R^2
esm3_sm_open_v1	**8.61**	**11.53**	**0.605**	**9.44**	**12.55**	**0.533**	0.83	1.02	0.073
esmc_300m	8.95	11.95	0.571	**9.47**	**12.54**	**0.528**	**0.52**	**0.59**	**0.043**
**ProtTransT5XLU50**	8.99	**11.95**	0.575	9.60	12.65	**0.524**	0.61	0.70	0.051
esmc_600m	9.48	12.92	0.547	9.87	13.39	0.513	**0.39**	**0.47**	**0.033**
ProtTransT5UniRef50	**8.79**	11.99	0.571	9.53	12.89	0.505	0.74	0.90	0.066
ProtTransT5BFD	8.94	12.22	0.554	9.58	13.03	0.493	0.64	0.81	0.061
ProtTransBertBFD	**8.50**	**11.20**	0.566	**9.49**	**12.24**	0.483	0.99	1.03	0.084
prime	9.98	13.13	0.483	10.03	13.22	0.476	**0.05**	**0.09**	**0.007**
ProtTransAlbertBFD	9.41	12.63	0.563	10.62	14.08	0.457	1.21	1.45	0.106
ProtTransXLNetUniRef100	9.31	12.59	0.556	10.55	14.02	0.450	1.24	1.43	0.106
FastText	9.40	12.50	**0.583**	11.57	15.25	0.380	2.17	2.75	0.202
SeqVec	9.05	12.18	0.558	11.12	14.55	0.372	2.08	2.38	0.186
CPCProt	10.34	13.29	0.550	12.40	15.85	0.363	2.06	2.57	0.187
Rostlab_ProstT5	9.44	12.53	0.529	11.34	14.74	0.347	1.90	2.21	0.181
Glove	9.56	12.53	0.522	11.41	14.84	0.330	1.85	2.31	0.192
Bepler	9.26	12.11	**0.580**	11.76	15.32	0.329	2.50	3.20	0.251
PLUSRNN	10.49	13.91	0.514	12.67	16.69	0.304	2.18	2.78	0.210
aaindex	9.09	12.04	0.504	11.22	14.41	0.291	2.14	2.37	0.214
composition	10.83	14.20	0.436	12.59	16.36	0.257	1.76	2.16	0.179
simple	9.76	13.50	0.408	12.19	16.01	0.168	2.42	2.52	0.240
physchem	9.79	12.91	0.518	13.91	17.49	0.118	4.11	4.57	0.400
auto	10.20	13.60	0.420	14.69	18.77	-	4.49	5.17	-

Table S3: Comparison of protein representations on the evaluation set. Sequence-level PLM embeddings were obtained via average pooling. For each representation, seven models (HistGradientBoosting, XGBoost, CatBoost, QuantileGBR, ElasticNet, SVR, and KernelRidge) were trained with hyperparameters optimised over 96 Optuna trials. Reported values are averaged across all models.

Protein Representation	R^2	MAE	MAE >90	MAE 70-90	MAE <30
**PT T5 XL U50**	**0.530**	**7.93**	**21.80**	15.32	**9.77**
Ankh3 XL	**0.514**	8.14	**20.54**	17.14	10.49
ESMC 600M	**0.512**	**8.10**	22.86	**13.64**	10.22
PT T5 U50	0.502	**7.98**	23.06	15.26	9.99
ESM2 650M	0.494	8.19	**19.67**	17.00	10.02
ESM2 3B	0.490	8.25	23.42	17.02	**9.73**
ESMC 300M	0.486	8.22	23.42	**14.88**	**9.77**
Prime	0.479	8.27	26.05	**13.09**	11.93
PT T5 BFD	0.453	8.22	28.70	16.07	10.51
ESM2 35M	0.450	13.19	26.57	23.68	13.81
Ankh Large	0.441	8.73	29.98	18.66	9.91
PT Bert BFD	0.431	8.60	28.65	16.89	11.25
Ankh3 Large	0.429	8.69	29.32	19.64	11.51
PT Albert BFD	0.420	8.31	26.35	18.95	10.65
PT XLNet U100	0.405	8.84	28.79	20.83	10.64
Ankh Base	0.400	13.40	35.19	22.09	13.53
PLUS RNN	0.368	9.21	29.12	20.18	11.12
SeqVec	0.345	9.51	33.11	20.86	12.27
*Simple	0.312	14.49	38.01	27.68	14.65
*AAIndex	0.291	14.45	41.10	29.62	14.65
GloVe	0.288	13.99	41.13	28.54	14.63
FastText	0.286	14.42	42.64	28.31	15.31
CPCProt	0.277	14.49	41.98	28.59	15.33
*Composition	0.276	14.26	41.62	27.53	15.00
Bepler	0.250	14.13	37.66	29.93	14.40
*Physicochemical	0.160	15.17	51.70	31.95	16.48
*Auto	0.033	16.33	56.57	38.95	16.40

Table S4: Dimensionality and generalization diagnostics for the protein representations evaluated in the feature-set experiment. Raw D is the number of `emb_` columns in the experiment CSVs. $\Delta\text{MAE} = \text{MAE}_{eval} - \text{MAE}_{train}$, and $\sigma_{\text{MAE,eval}}$ measures sensitivity across model families, not repeated-seed variability.

Protein representation	Family	Raw D	N_{train}	D/N_{train}	ΔMAE	$\sigma_{\text{MAE,eval}}$
PT Albert BFD	Transformer	4,096	6,339	0.646	5.91	0.05
Ankh3 XL	Transformer	2,560	6,338	0.404	5.94	0.12
ESM2 3B	Transformer	2,560	6,338	0.404	5.47	0.12
Ankh Large	Transformer	1,536	6,338	0.242	5.32	0.19
Ankh3 Large	Transformer	1,536	6,338	0.242	5.89	0.15
ESM2 650M	Transformer	1,280	6,338	0.202	5.42	0.07
Prime	Transformer	1,280	6,338	0.202	3.74	0.08
ESMC 600M	Transformer	1,152	6,338	0.182	5.76	0.02
PT Bert BFD	Transformer	1,024	6,338	0.162	6.49	0.15
PT T5 BFD	Transformer	1,024	6,338	0.162	6.05	0.16
PT T5 U50	Transformer	1,024	6,339	0.162	6.14	0.08
PT T5 XL U50	Transformer	1,024	6,338	0.162	5.55	0.03
PT XLNet U100	Transformer	1,024	6,338	0.162	7.03	0.04
ESMC 300M	Transformer	960	6,338	0.151	6.36	0.02
Ankh Base	Transformer	768	6,338	0.121	4.25	10.87
ESM2 35M	Transformer	480	6,338	0.076	4.10	11.10
PLUS RNN	Other	1,024	6,338	0.162	6.34	0.07
SeqVec	Other	1,024	6,338	0.162	6.43	0.16
CPCProt	Other	512	6,338	0.081	4.66	10.53
FastText	Other	512	6,338	0.081	4.71	10.56
GloVe	Other	512	6,338	0.081	4.34	10.76
Bepler	Other	121	6,338	0.019	4.29	10.73
*AAIndex	Physicochemical	553	6,338	0.087	4.27	10.71
*Composition	Physicochemical	420	6,338	0.066	4.75	10.66
*Physicochemical	Physicochemical	73	6,338	0.012	4.08	10.16
*Simple	Physicochemical	40	6,338	0.006	4.28	10.66
*Auto	Physicochemical	24	6,338	0.004	4.73	9.89

Table S5: Comparison of resampling strategies. $MAE_{<25 \text{ or } >60}$ is calculated for observations with $T_{opt} < 25\,°C$ or $T_{opt} > 60\,°C$.

Resampling Method	R^2	MAE	$MAE_{<25 \text{ or } >60}$	MAE >60	MAE <25
Tomek Links	**0.456**	**8.43**	14.96	15.43	14.21
ADASYN	**0.457**	**8.48**	14.75	15.16	14.08
EXT TomekLinks	0.446	**8.50**	14.33	14.36	14.28
**Baseline**	**0.447**	8.53	14.70	14.82	14.50
EXT KNNOR	0.430	8.66	13.91	13.63	14.33
KNNOR	0.428	8.67	14.19	13.97	14.53
EXT SMOGN	0.414	8.78	13.37	13.08	**13.80**
EXT SMOTER	0.412	8.81	13.40	13.13	13.81
EXT ADASYN	0.417	8.83	13.39	13.40	**13.35**
EXT WERCS	0.398	8.88	13.43	12.87	14.30
EXT WERCS-GN	0.397	8.91	13.39	12.77	14.33
IHT	0.396	8.93	19.38	21.54	16.00
WERCS-GN	0.399	8.99	13.53	13.02	14.31
WERCS	0.397	9.00	13.63	13.20	14.30
Cluster Centroids	0.405	9.02	16.00	16.40	15.38
SMOGN	0.383	9.04	13.53	12.78	14.70
SMOTER	0.382	9.05	13.62	12.87	14.78
**EXT ENN**	0.358	9.19	**12.90**	**12.29**	13.85
CNN	0.379	9.21	15.97	16.11	15.75
EXT Random Over	0.353	9.22	13.26	12.65	14.20
EXT Cluster Centroids	0.365	9.24	13.28	12.76	14.07
EXT IHT	0.341	9.25	13.18	**12.13**	14.80
EXT CNN	0.354	9.29	13.10	12.48	14.04
EXT Random Under	0.351	9.29	**13.05**	**12.36**	14.12
ENN	0.280	9.66	21.90	25.62	16.09
EXT OSS	0.317	9.82	14.46	14.41	14.52
EXT NearMiss	0.243	10.23	**13.09**	12.50	14.00
NearMiss	0.259	10.35	17.80	17.39	18.42
OSS	0.235	10.48	13.12	14.01	**11.71**

Table S6: Comparison of all trained models on the eval set. $\mathrm{MAE}_{<20 \text{ or } >60}$ is calculated for observations with $T_{\text{opt}} < 20\,°\text{C}$ or $T_{\text{opt}} > 60\,°\text{C}$.

	Training		Eval		|Δ Train – Eval|		Eval MAE (ranges)		
Model	R^2	MAE	R^2	MAE	ΔR^2	ΔMAE	> 90	< 30	$\mathrm{MAE}_{<20 \text{ or } >60}$
GPR	**1.000**	**0.00**	**0.560**	**7.66**	0.440	7.66	21.41	9.11	16.27
KernelRidge	**0.941**	**2.41**	**0.559**	**7.50**	0.382	5.09	17.17	8.38	15.80
BaggingMLP	0.832	4.55	**0.534**	**7.82**	0.298	3.27	17.24	7.17	12.50
ResNetMLP	**0.915**	**3.34**	0.524	7.84	0.391	4.50	14.99	6.59	14.42
MLP	0.708	6.12	0.510	8.22	0.198	2.10	16.68	9.53	13.22
RandomForest	0.764	5.56	0.489	8.43	0.276	2.87	20.40	8.56	14.82
ARDRegression	0.582	7.47	0.489	8.37	0.094	0.90	23.48	8.95	16.89
RealMLP	0.758	5.48	0.486	8.24	0.272	2.76	17.20	6.42	12.78
ExtraTrees	0.742	5.78	0.471	8.61	0.271	2.83	19.15	8.04	13.94
WideDeep	0.847	4.53	0.471	8.46	0.376	3.93	15.79	6.92	13.20
SparseMLP	0.830	4.71	0.465	8.46	0.365	3.75	16.23	7.51	13.35
PLS	0.595	7.40	0.458	8.63	0.137	1.23	22.87	8.75	16.49
TabM	0.795	4.63	0.453	8.39	0.342	3.75	17.73	7.56	12.70
OMP	0.491	8.28	0.452	8.74	0.039	0.46	25.91	9.74	17.99
**CatBoost**	0.708	5.97	0.449	8.84	0.259	2.86	**13.28**	7.42	12.39
HistGradientBoosting	0.664	6.57	0.446	8.90	0.218	2.33	16.07	7.59	12.69
DANets	0.548	7.99	0.444	8.92	0.104	0.92	15.99	6.87	12.95
PassiveAggressive	0.572	7.17	0.442	8.56	0.130	1.39	21.58	8.28	16.21
BaselineMLP	0.738	6.03	0.438	8.64	0.300	2.61	13.52	6.75	12.49
Node	0.558	7.82	0.437	8.93	0.121	1.11	18.08	**5.66**	12.75
**BaggingSVR**	0.651	6.39	0.436	9.05	0.215	2.65	**13.19**	7.22	12.12
GANDALF	0.573	7.55	0.436	8.85	0.137	1.30	19.09	7.65	12.62
LassoNet	0.594	7.41	0.436	8.85	0.159	1.44	14.42	7.73	11.98
TabR	0.681	6.42	0.435	8.68	0.246	2.26	15.74	6.18	12.10
BaggingDecisionTree	0.629	7.21	0.429	9.06	0.200	1.86	18.10	8.00	13.75
XGBoost	0.654	6.80	0.420	9.14	0.234	2.34	13.86	7.11	12.23
GradientBoosting	0.694	5.92	0.419	9.08	0.274	3.16	13.60	6.80	12.30
AdaBoost	0.460	8.77	0.419	9.09	0.041	**0.32**	21.29	12.03	15.07
SVR	0.634	6.00	0.418	9.13	0.216	3.13	13.58	6.92	12.54
FTTransformer	0.432	8.60	0.417	8.78	**0.015**	**0.18**	19.87	8.44	14.13
GatedMLP	0.748	5.93	0.416	8.99	0.332	3.06	14.95	7.20	13.15
RANSAC	0.586	6.96	0.409	8.90	0.177	1.94	21.05	7.98	16.49
TabNetInspired	0.797	5.10	0.408	9.04	0.388	3.93	21.37	8.05	17.20
AttentionMLP	0.738	6.19	0.402	9.37	0.336	3.18	15.81	6.38	13.86
Hopular	0.565	7.60	0.399	9.08	0.165	1.48	15.70	6.67	11.95
BaggingSVRExtreme	0.593	6.92	0.386	9.51	0.208	2.59	13.51	7.37	12.16
BaggingDecisionTreeExtreme	0.584	7.51	0.375	9.56	0.209	2.05	16.71	7.60	12.97
BaggingMLPExtreme	0.626	6.85	0.366	9.54	0.260	2.69	13.69	**5.59**	**11.23**
RTDLResNet	0.480	8.24	0.356	9.31	0.124	1.06	14.94	**5.36**	**11.18**
TabNet	0.440	8.68	0.351	9.35	0.088	0.67	22.51	6.18	15.47
BayesianRidge	0.387	9.34	0.349	9.74	**0.038**	0.40	15.55	7.65	12.07
SAINT	0.367	9.29	0.342	9.55	**0.025**	**0.26**	27.38	6.74	17.81
Ridge	0.386	9.36	0.318	10.03	0.068	0.67	15.20	7.45	11.99
LinearRegression	0.258	10.33	0.093	11.56	0.165	1.23	15.61	9.35	13.03
ElasticNet	0.324	9.74	0.066	11.94	0.259	2.20	14.81	9.37	13.91
Quantile	0.158	10.29	0.064	11.58	0.094	1.29	**10.74**	10.50	**10.52**
DecisionTree	0.148	10.81	-0.060	11.92	0.208	1.11	21.08	11.18	16.22
Huber	0.163	10.52	-0.195	13.47	0.359	2.95	16.67	10.86	15.03
SNN	-0.172	12.96	-0.518	14.72	0.346	1.76	19.93	14.98	24.45

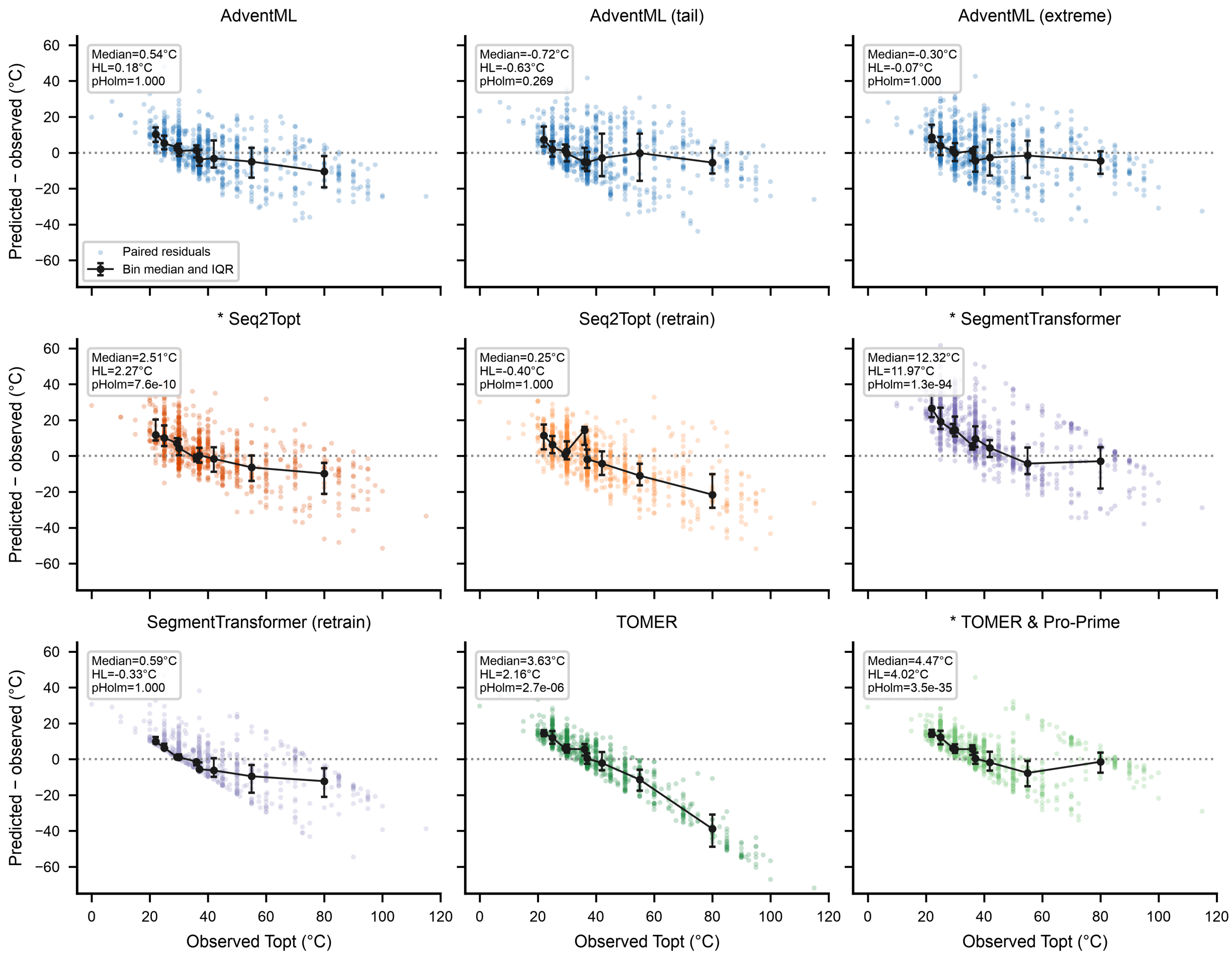

Figure S1: Prediction residuals as a function of observed optimal temperature. Predicted-minus-observed residuals for AdventML and all benchmarked models on the held-out test set. Positive residuals indicate overprediction. Black points and error bars show the median and interquartile range within ten equal-frequency bins of observed T_{opt}. Each panel reports the overall residual median, Hodges-Lehmann pseudomedian bias (HL), and Holm-adjusted p-value from a two-sided Wilcoxon signed-rank test. An asterisk preceding a model name indicates overlap between its original training data and the test set.

Table S7: Comparison of SOTA models on the held-out test set.

Model	R^2	MAE	RMSE	MAE >90	MAE 70-90	MAE <30
**AdventML**	**0.646**	**7.58**	**10.14**	16.78	11.32	**8.04**
SegmentTransformer (retrain)	**0.606**	**7.68**	**10.70**	22.41	12.47	9.47
TOMER & Pro-Prime	**0.605**	**8.28**	**10.72**	**8.58**	**8.13**	13.75
AdventML (tail)	0.558	8.45	11.33	**12.33**	**9.48**	**7.42**
AdventML (extreme)	0.536	8.85	11.61	**12.77**	**9.16**	**8.78**
Seq2Topt	0.455	9.36	12.59	18.12	13.44	12.88
Seq2Topt (retrain)	0.443	9.57	12.73	27.05	20.56	9.18
TOMER	0.079	11.31	16.37	54.17	35.12	13.49
SegmentTransformer	-0.190	15.29	18.60	13.11	12.16	23.16

Table S8: Pairwise MAE >70 significance matrix (paired bootstrap, B = 100,000, BH-corrected $\alpha = 0.05$). > row significantly better than column; < significantly worse; = not significant. Models ordered by MAE >70 (ascending). Only p-values higher than 0.001 are shown.

Model	*TOMER & Pro-Prime*	*AdventML (extreme)*	*AdventML (tail)*	*SegmentTransformer*	*AdventML*	*Seq2Topt*	*SegmentTransformer (retrain)*	*Seq2Topt (retrain)*	*TOMER*
**TOMER & Pro-Prime**	-	>	>	>	>	>	>	>	>
**AdventML (extreme)**	<	-	= (0.70)	= (0.21)	>	>	>	>	>
**AdventML (tail)**	<	= (0.70)	-	= (0.26)	>	>	>	>	>
**SegmentTransformer**	<	= (0.21)	= (0.26)	-	= (0.23)	> (0.01)	>	>	>
**AdventML**	<	<	<	= (0.23)	-	> (0.04)	>	>	>
**Seq2Topt**	<	<	<	< (0.01)	< (0.04)	-	= (0.68)	>	>
**SegmentTransformer (retrain)**	<	<	<	<	<	= (0.68)	-	>	>
**Seq2Topt (retrain)**	<	<	<	<	<	<	<	-	>
**TOMER**	<	<	<	<	<	<	<	<	-

Table S9: Pairwise MAE >90 significance matrix (paired bootstrap, B = 100,000, BH-corrected $\alpha = 0.05$). > row significantly better than column; < significantly worse; = not significant. Models ordered by MAE >90 (ascending). Only p-values higher than 0.001 are shown.

Model	*TOMER & Pro-Prime*	*AdventML (tail)*	*AdventML (extreme)*	*SegmentTransformer*	*AdventML*	*Seq2Topt*	*SegmentTransformer (retrain)*	*Seq2Topt (retrain)*	*TOMER*
**TOMER & Pro-Prime**	-	= (0.27)	= (0.07)	= (0.06)	>	>	>	>	>
**AdventML (tail)**	= (0.27)	-	= (0.15)	= (0.50)	>	= (0.13)	>	>	>
**AdventML (extreme)**	= (0.07)	= (0.15)	-	= (0.81)	= (0.19)	= (0.25)	>	>	>
**SegmentTransformer**	= (0.06)	= (0.50)	= (0.81)	-	= (0.61)	= (0.52)	> (0.03)	>	>
**AdventML**	<	<	= (0.19)	= (0.61)	-	= (0.66)	>	>	>
**Seq2Topt**	<	= (0.13)	= (0.25)	= (0.52)	= (0.66)	-	= (0.27)	= (0.05)	>
**SegmentTransformer (retrain)**	<	<	<	< (0.03)	<	= (0.27)	-	= (0.15)	>
**Seq2Topt (retrain)**	<	<	<	<	<	= (0.05)	= (0.15)	-	>
**TOMER**	<	<	<	<	<	<	<	<	-

Table S10: Pairwise MAE <30 significance matrix (paired bootstrap, $B = 100{,}000$, BH-corrected $\alpha = 0.05$). > row significantly better than column; < significantly worse; = not significant. Models ordered by MAE <30 (ascending). Only p-values higher than 0.001 are shown.

Model	*AdventML (tail)*	*AdventML*	*AdventML (extreme)*	*Seq2Topt (retrain)*	*SegmentTransformer (retrain)*	*Seq2Topt*	*TOMER*	*TOMER & Pro-Prime*	*SegmentTransformer*
**AdventML (tail)**	-	> (0.05)	>	>	>	>	>	>	>
**AdventML**	< (0.05)	-	= (0.06)	> (0.03)	>	>	>	>	>
**AdventML (extreme)**	<	= (0.06)	-	= (0.55)	= (0.18)	>	>	>	>
**Seq2Topt (retrain)**	<	< (0.03)	= (0.55)	-	= (0.55)	>	>	>	>
**SegmentTransformer (retrain)**	<	<	= (0.18)	= (0.55)	-	>	>	>	>
**Seq2Topt**	<	<	<	<	<	-	= (0.32)	= (0.16)	>
**TOMER**	<	<	<	<	<	= (0.32)	-	= (0.13)	>
**TOMER & Pro-Prime**	<	<	<	<	<	= (0.16)	= (0.13)	-	>
**SegmentTransformer**	<	<	<	<	<	<	<	<	-

Table S11: Pairwise R^2 significance matrix (paired bootstrap, $B = 100{,}000$, BH-corrected $\alpha = 0.05$). > row significantly better than column; < significantly worse; = not significant. Models ordered by R^2 (descending). Only p-values higher than 0.001 are shown.

Model	*AdventML*	*SegmentTransformer (retrain)*	*TOMER & Pro-Prime*	*AdventML (tail)*	*AdventML (extreme)*	*Seq2Topt*	*Seq2Topt (retrain)*	*TOMER*	*SegmentTransformer*
**AdventML**	-	> (0.02)	> (0.03)	>	>	>	>	>	>
**SegmentTransformer (retrain)**	< (0.02)	-	= (0.95)	> (0.04)	>	>	>	>	>
**TOMER & Pro-Prime**	< (0.03)	= (0.95)	-	> (0.04)	>	>	>	>	>
**AdventML (tail)**	<	< (0.04)	< (0.04)	-	= (0.19)	>	>	>	>
**AdventML (extreme)**	<	<	<	= (0.19)	-	> (0.02)	>	>	>
**Seq2Topt**	<	<	<	<	< (0.02)	-	= (0.74)	>	>
**Seq2Topt (retrain)**	<	<	<	<	<	= (0.74)	-	>	>
**TOMER**	<	<	<	<	<	<	<	-	>
**SegmentTransformer**	<	<	<	<	<	<	<	<	-

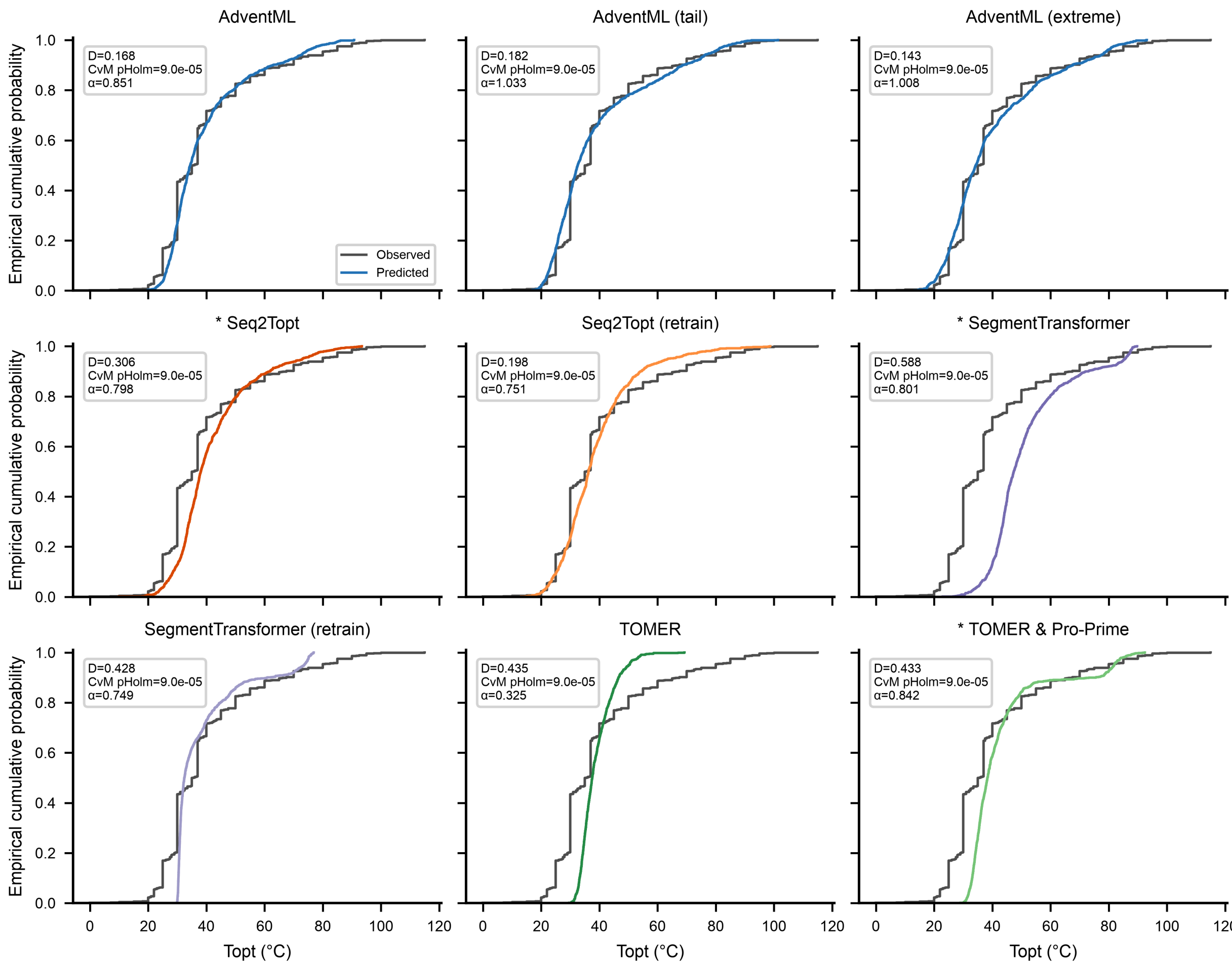

Figure S2: Observed and predicted marginal distributions for all models. Empirical cumulative distribution functions of observed and predicted T_{opt} values for the held-out test set. Each panel reports the maximum ECDF separation (D), the Holm-adjusted paired Cramér-von Mises p-value, and the variability ratio $\alpha = \mathrm{SD(predicted)/SD(observed)}$. These comparisons assess agreement between marginal distributions rather than the accuracy of individual paired predictions. An asterisk preceding a model name indicates overlap between its original training data and the test set.

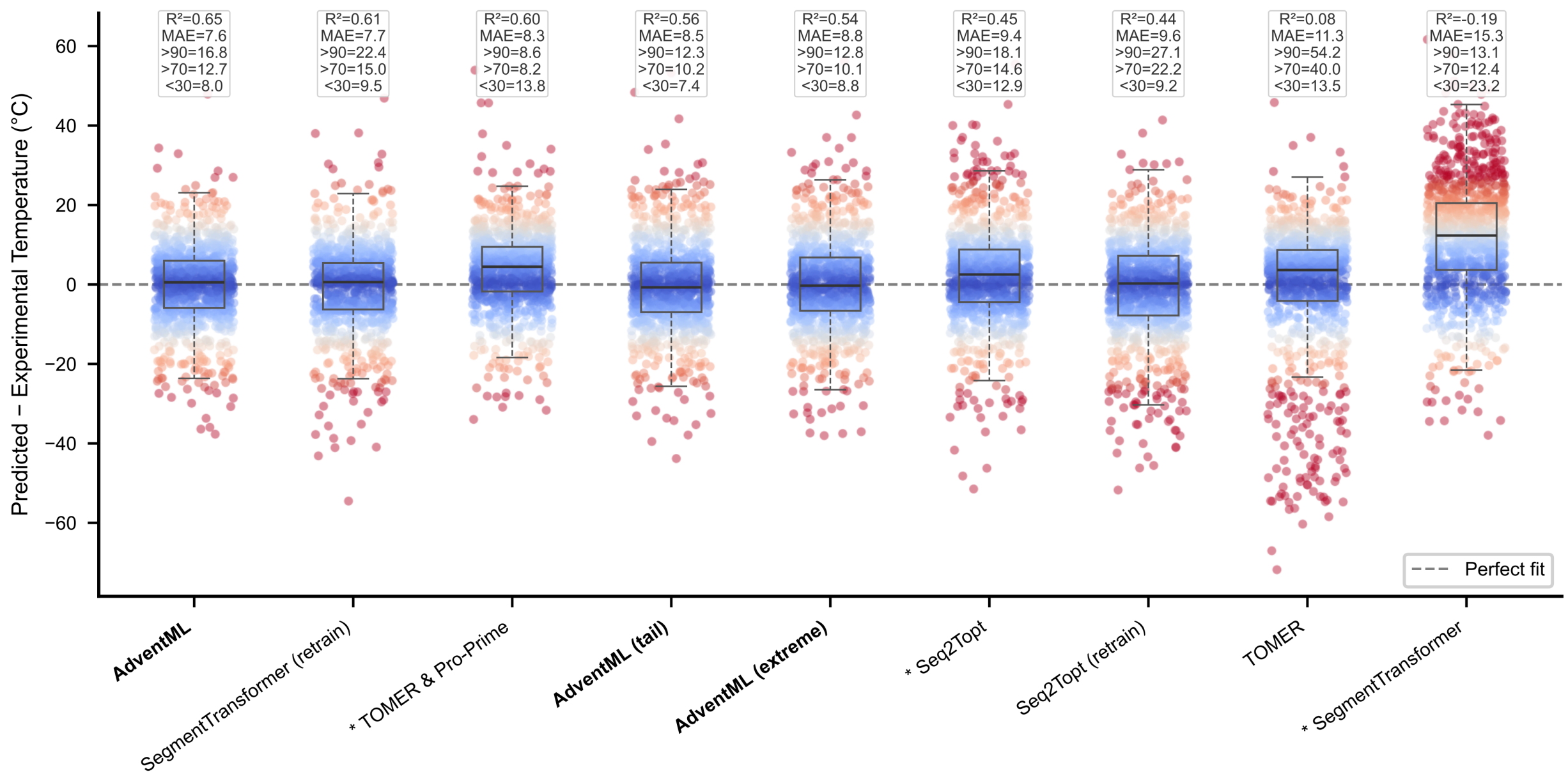

Figure S3: Prediction residual distributions across models. Strip plot of prediction residuals for AdventML and all benchmarked models on the held-out test set. Positive values correspond to predictions exceeding the experimental T_{opt}. Asterisks (*) denote models whose training data overlaps with the test set.

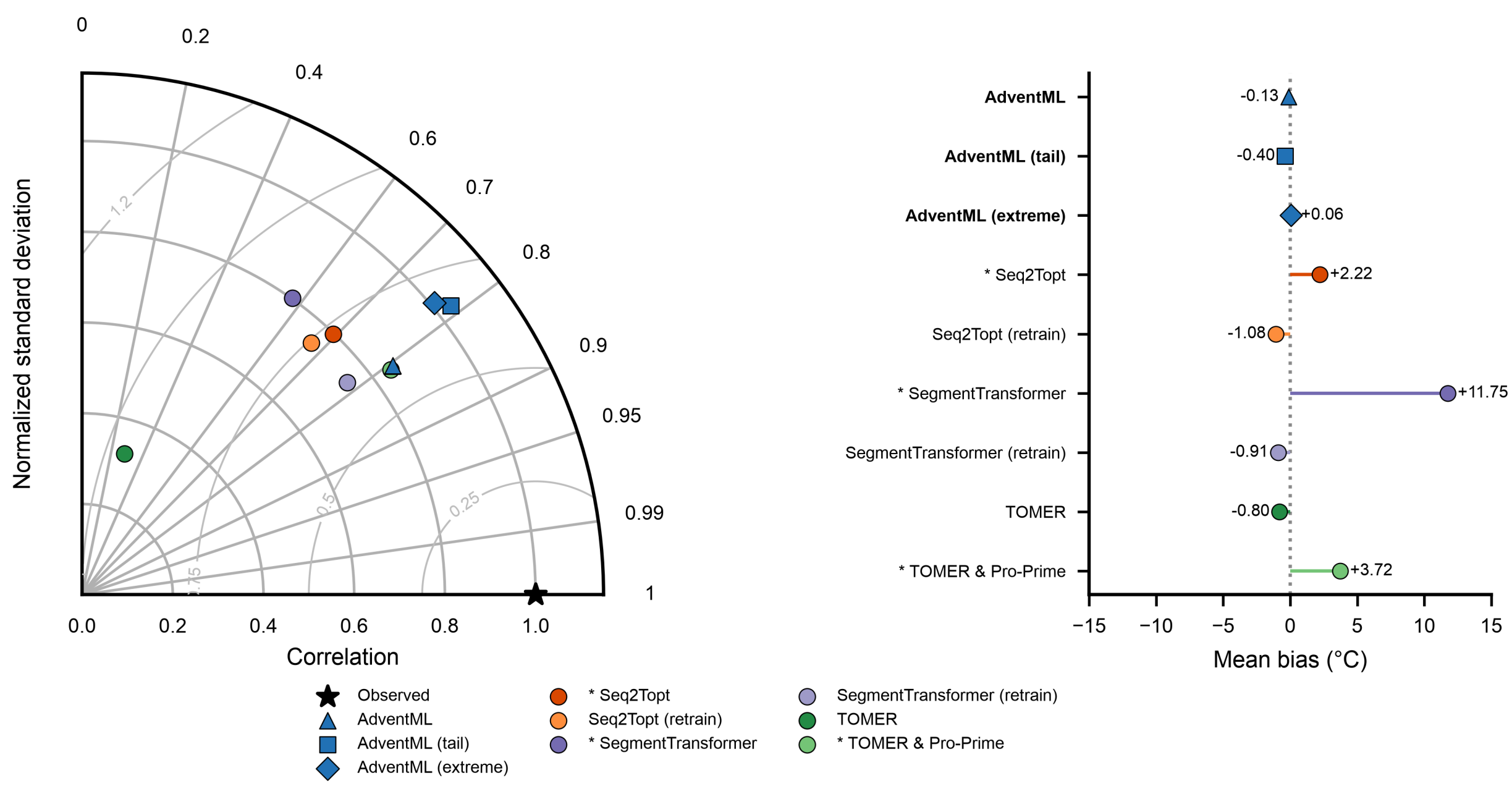

Figure S4: Taylor-diagram comparison of model performance and mean bias. The normalized Taylor diagram summarizes the Pearson correlation, normalized standard deviation α = SD(predicted)/SD(observed), and normalized centred root-mean-square error for each model. The star at $r = 1$ and $\alpha = 1$ represents perfect agreement with the observations, and the grey contours denote normalized centred RMSE. The adjacent panel shows the mean prediction bias, calculated as predicted minus observed T_{opt}; positive values indicate overprediction. An asterisk preceding a model name indicates overlap between its original training data and the test set.

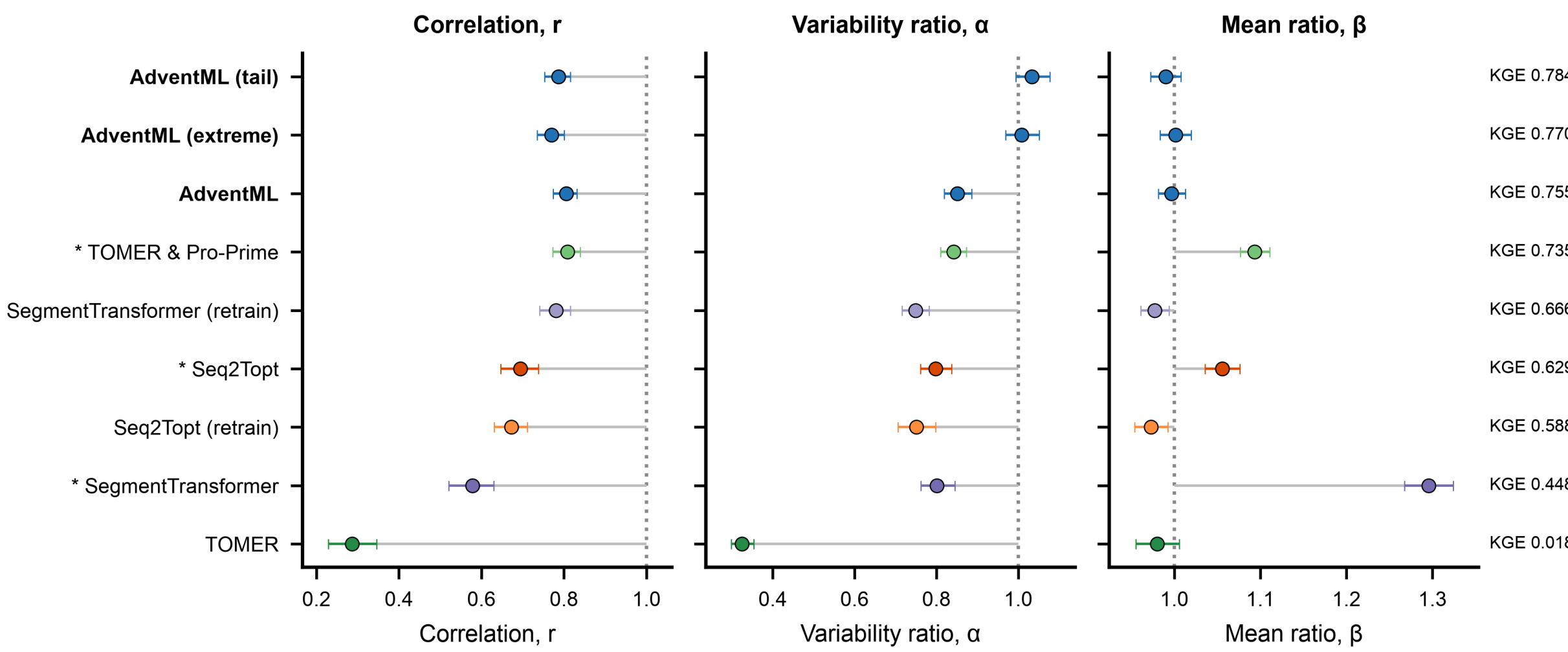

Figure S5: Kling-Gupta efficiency components for the evaluated models. Point estimates and paired-protein 95% percentile-bootstrap intervals are shown for the Pearson correlation (r), variability ratio $\alpha = \text{SD(predicted)}/\text{SD(observed)}$, and mean ratio $\beta = \text{mean(predicted)}/\text{mean(observed)}$. Models are ordered by decreasing Kling-Gupta efficiency, $\text{KGE} = 1 - \sqrt{(r-1)^2 + (\alpha-1)^2 + (\beta-1)^2}$, with the corresponding KGE value reported at the right. The vertical reference line at one denotes the ideal value for each component. Because degrees Celsius have an arbitrary zero, β and KGE depend on the temperature scale origin; conventional KGE quality thresholds should therefore not be applied directly. An asterisk preceding a model name indicates overlap between its original training data and the test set.

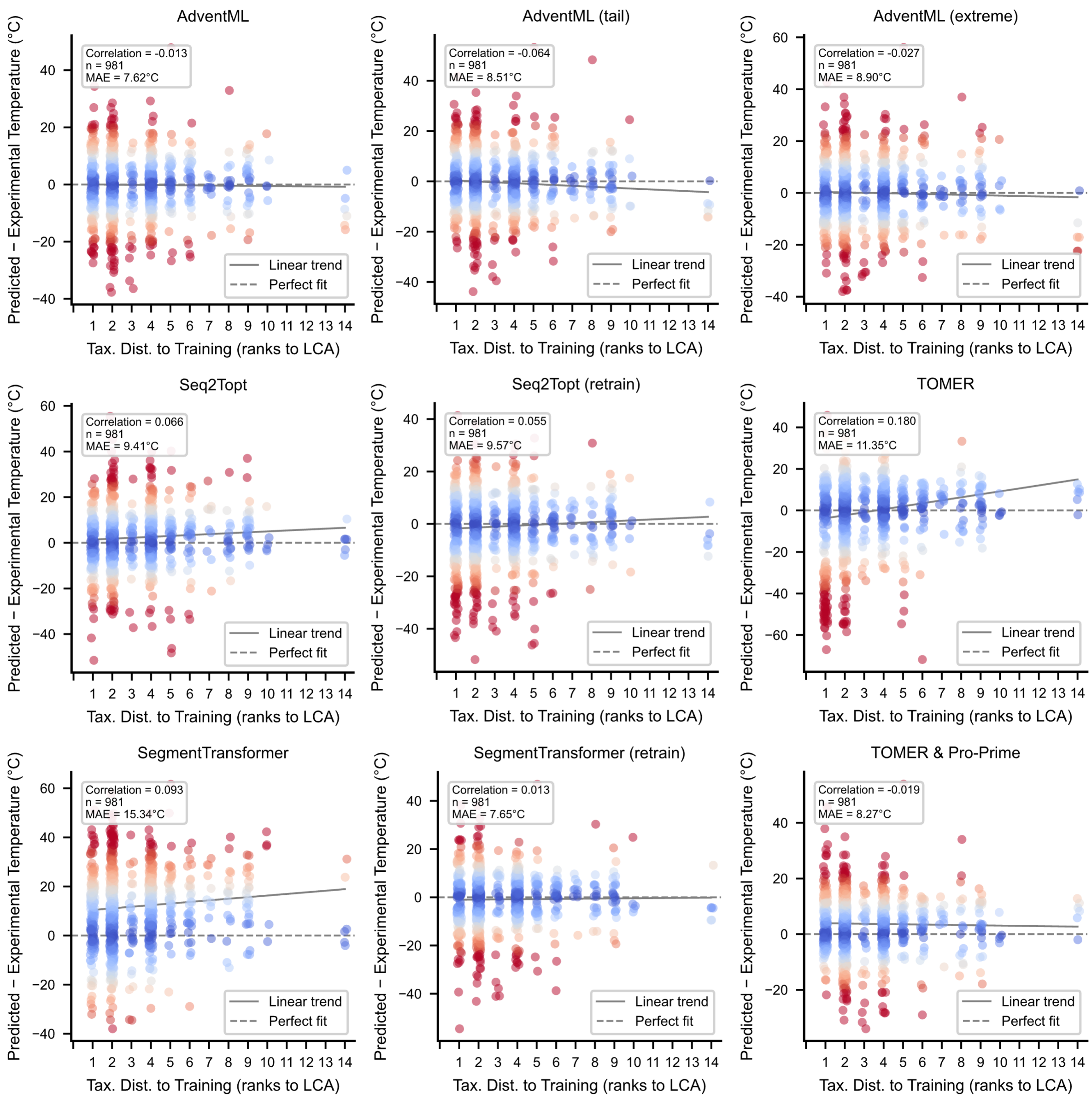

Figure S6: Prediction error stratified by phylogenetic distance. Model performance as a function of phylogenetic distance between test and training sequences. Phylogenetic distance showed by branch count between test observation, closest training sample and last common ancestor. Positive values correspond to predictions exceeding the experimental T_{opt}.

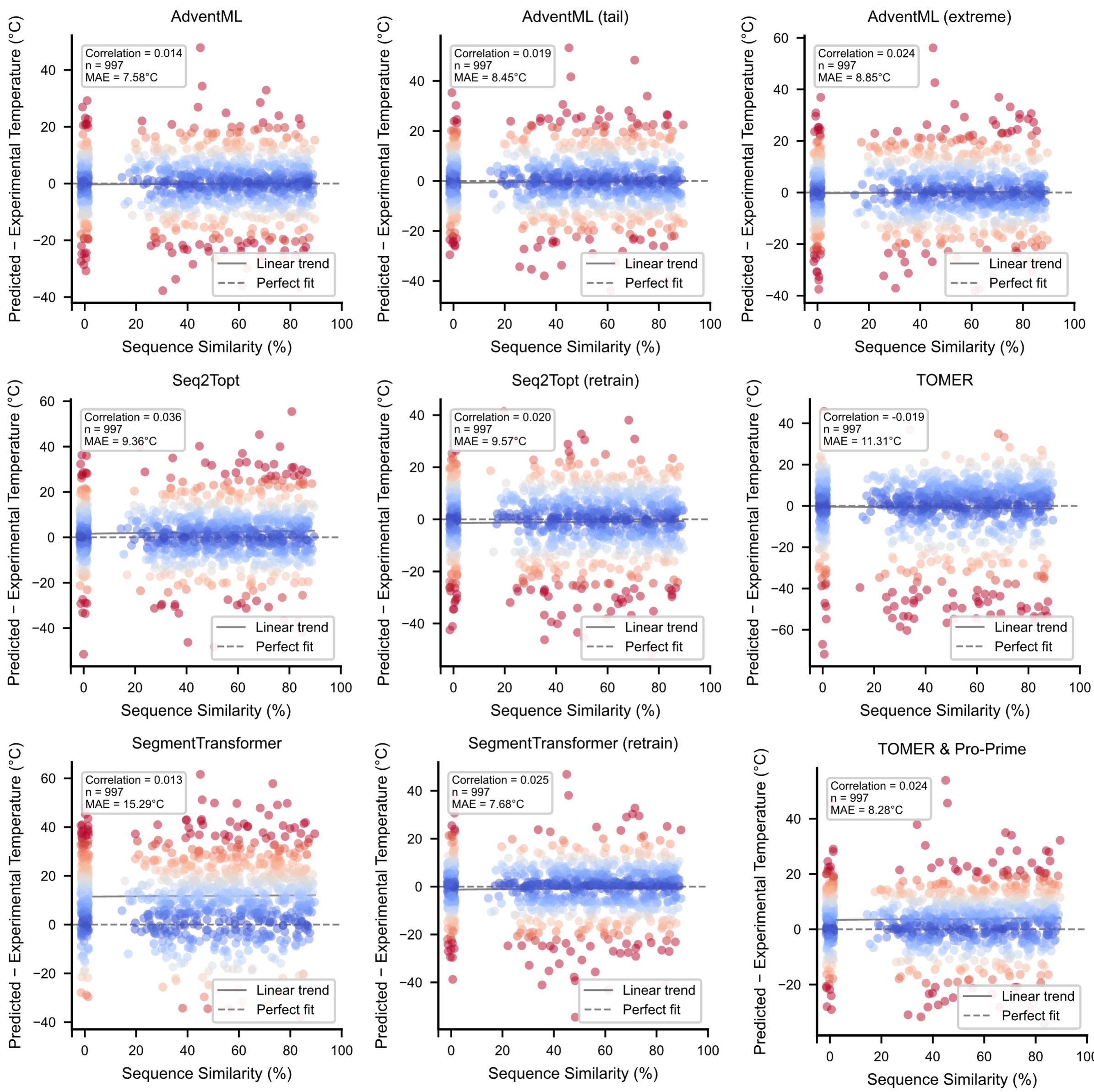

Figure S7: Prediction performance as a function of sequence similarity. Model accuracy stratified by maximum sequence identity between each test sequence and the training set. Positive values correspond to predictions exceeding the experimental T_{opt}.

Table S12: Paired residual-location and marginal-distribution comparisons between experimental and predicted protein optimum temperatures.

A. Wilcoxon signed-rank comparisons

Model	HL shift (95% CI), °C	V	p_{Holm}
AdventML	$+0.183$ [-0.398, $+0.756$]	254,420	1.000
AdventML (tail)	-0.633 [-1.276, $+0.010$]	231,212	0.269
AdventML (extreme)	-0.074 [-0.767, $+0.608$]	246,749	1.000
Seq2Topt	$+2.271$ [$+1.597$, $+2.953$]	307,458	7.57×10^{-10}
Seq2Topt (retrain)	-0.397 [-1.150, $+0.345$]	239,299	1.000
SegmentTransformer	$+11.973$ [$+11.121$, $+12.833$]	437,389	1.31×10^{-94}
SegmentTransformer (retrain)	-0.334 [-0.991, $+0.308$]	239,514	1.000
TOMER	$+2.160$ [$+1.388$, $+2.898$]	294,668.5	2.67×10^{-6}
TOMER & Pro-Prime	$+4.025$ [$+3.448$, $+4.590$]	362,147	3.51×10^{-35}

B. Paired marginal-distribution comparisons

Model	Paired CvM	Paired KS: D	Paired KS: T_D, °C
AdventML	6.262	0.168	30.0
AdventML (tail)	2.869	0.182	30.0
AdventML (extreme)	2.920	0.143	30.0
Seq2Topt	25.976	0.306	30.0
Seq2Topt (retrain)	9.029	0.198	30.0
SegmentTransformer	128.038	0.588	37.5
SegmentTransformer (retrain)	38.611	0.428	30.0
TOMER	51.455	0.435	31.0
TOMER & Pro-Prime	51.044	0.433	30.0

All analyses used the same $n = 997$ held-out protein pairs. Residuals were defined as predicted minus observed T_{opt}; positive Hodges-Lehmann (HL) shifts indicate overprediction and negative shifts indicate underprediction. Brackets give the unadjusted 95% confidence interval for the HL shift. V is the two-sided Wilcoxon signed-rank test statistic. CvM summarizes differences across the observed and predicted cumulative distributions. KS D measures the size of the largest gap between these cumulative distributions, and KS T_D is the temperature where that gap occurs. Smaller CvM and D values indicate closer distributional agreement; T_D identifies the location of the largest discrepancy rather than its severity. CvM and KS p-values were estimated using bootstrap ($B = 100{,}000$). Holm adjustment was applied separately across the nine models within each test family. HL shifts were small for all AdventML variants ($|\mathrm{HL}| < 1°\mathrm{C}$), with no significant directional bias detected. AdventML tail had the lowest CvM statistic (2.869), closely followed by AdventML extreme (2.920), indicating the closest overall distributional agreement. AdventML extreme had the smallest maximum distributional gap ($D = 0.143$, at $30°\mathrm{C}$). Nevertheless, distributional differences remained statistically significant for every model.

Table S13: Fit and bias metrics on the aligned held-out test set ($n = 997$). Values are point estimates with paired-protein 95% percentile-bootstrap confidence intervals ($B = 10{,}000$).

A. Fit and error metrics

Model	R^2 (= NSE)	MAPE (%)	RMSE (°C)
AdventML	**0.646** **[0.592, 0.691]**	20.900 [19.460, 22.515]	**10.138** **[9.562, 10.712]**
AdventML (tail)	0.558 [0.479, 0.621]	23.191 [21.661, 24.843]	11.332 [10.699, 11.990]
AdventML (extreme)	0.536 [0.453, 0.603]	24.897 [23.308, 26.605]	11.609 [10.979, 12.244]
* Seq2Topt	0.455 [0.370, 0.528]	27.551 [25.509, 29.870]	12.592 [11.854, 13.324]
Seq2Topt (retrain)	0.443 [0.378, 0.499]	25.374 [23.753, 27.208]	12.726 [12.020, 13.436]
* SegmentTransformer	-0.190 [-0.385, -0.034]	48.833 [46.006, 51.853]	18.598 [17.841, 19.358]
SegmentTransformer (retrain)	0.606 [0.546, 0.656]	**20.720** **[19.274, 22.357]**	10.703 [9.988, 11.424]
TOMER	0.079 [0.041, 0.118]	29.023 [27.153, 31.105]	16.366 [15.232, 17.476]
* TOMER & Pro-Prime	0.605 [0.537, 0.662]	25.648 [23.690, 27.722]	10.720 [10.133, 11.333]

B. Efficiency and bias metrics

Model	KGE	PBIAS (%)
AdventML	0.755 [0.725, 0.782]	0.315 [-1.287, 1.855]
AdventML (tail)	**0.784** **[0.748, 0.813]**	1.006 [-0.763, 2.767]
AdventML (extreme)	0.770 [0.733, 0.800]	-**0.145** **[-1.950, 1.665]**
* Seq2Topt	0.629 [0.584, 0.670]	-5.573 [-7.633, -3.638]
Seq2Topt (retrain)	0.588 [0.541, 0.631]	2.726 [0.777, 4.674]
* SegmentTransformer	0.448 [0.397, 0.491]	-29.550 [-32.473, -26.759]
SegmentTransformer (retrain)	0.666 [0.629, 0.698]	2.283 [0.679, 3.856]
TOMER	0.018 [-0.034, 0.071]	2.005 [-0.576, 4.449]
* TOMER & Pro-Prime	0.735 [0.701, 0.763]	-9.346 [-11.094, -7.666]

MAPE excludes the one observation with zero T_{opt} ($n = 996$). Positive PBIAS indicates aggregate underprediction; negative PBIAS indicates overprediction. Bold marks the best point estimate (PBIAS closest to zero). An asterisk denotes overlap between a model's original training data and the test set.

Table S14: Sequence-length statistics for the BRENDA and TOMER datasets. Lengths are measured in amino-acid residues; n is the number of sequences, and mode counts are shown in parentheses.

	BRENDA			TOMER		
Statistic	Train/ validation	Held-out test	Total	Training	Test	Total
n	9055	997	10052	2333	584	2917
Mean	471.3	476.7	471.8	456.8	449.0	455.3
SD	298.2	312.4	299.7	262.2	238.9	257.7
Median	413.0	406.0	412.0	394.0	402.0	395.0
Q1-Q3	311.0-549.0	312.0-546.0	311.0-549.0	297.0-538.0	296.8-535.2	297.0-537.0
Min-Max	61-7096	66-3391	61-7096	36-3332	34-2072	34-3332
Mode (n)	250 (36)	278 (8)	250 (38)	250 (22)	340 (6)	250 (23)
Skew	5.88	4.27	5.70	2.73	1.87	2.60
Excess kurtosis	80.22	28.92	74.26	14.43	6.17	13.31

Models and hyperparameter settings

Table S15: Protein representations names and descriptions.

Abbreviation	Full Written Name / Description	Category
PT T5 XL U50	ProtTrans T5-XL-U50	Transformer
PT T5 U50	ProtTrans T5-UniRef50	Transformer
PT T5 BFD	ProtTrans T5-BFD	Transformer
PT Bert BFD	ProtTrans BERT-BFD	Transformer
PT Albert BFD	ProtTrans ALBERT-BFD	Transformer
PT XLNet U100	ProtTrans XLNet-UniRef100	Transformer
ProstT5	RostLab ProstT5	Transformer
ESM2 3B	ESM-2 (3 Billion parameters)	Transformer
ESM2 650M	ESM-2 (650 Million parameters)	Transformer
ESM2 35M	ESM-2 (35 Million parameters)	Transformer
ESM2	ESM-2 (Base/Default version)	Transformer
ESM1b	ESM-1b	Transformer
ESMC 600M	ESM-C (600 Million parameters)	Transformer
ESMC 300M	ESM-C (300 Million parameters)	Transformer
ESM3 SM	ESM-3 Small Open v1	Transformer
Ankh3 XL	Ankh3 Extra Large	Transformer
Ankh3 Large	Ankh3 Large	Transformer
Ankh Large	Ankh Large	Transformer
Ankh Base	Ankh Base	Transformer
Prime	AI4Protein/Prime	Transformer
PLUS RNN	PLUS Recurrent Neural Network	Other Learned
SeqVec	Sequence Vector (SeqVec)	Other Learned
GloVe	Global Vectors for Word Representation	Other Learned
FastText	FastText	Other Learned
CPCProt	Contrastive Predictive Coding for Proteins	Other Learned
Bepler	Bepler & Berger Embeddings	Other Learned
*Physicochemical	**Comprehensive Physicochemical Properties:** Includes molecular weight, hydrophobicity, isoelectric point, normalized van der Waals volume, polarizability, generic structural descriptors, and solvent accessibility metrics.	Physicochemical
*Composition	**Amino Acid & PAAC:** Amino acid frequency composition (20 features) and Pseudo-amino acid composition (PAAC), which incorporates sequence order information.	Physicochemical
*AAIndex	**AAindex-derived Properties:** Direct numerical indices of all physicochemical and biological properties of amino acids (AAindex database).	Physicochemical
*Auto	**Autocorrelation Descriptors:** Moreau-Broto, Moran, and Geary autocorrelation metrics that measure the distribution of amino acid properties along the protein sequence.	Physicochemical
*Simple	**Simple Descriptors & CTD:** Conjoint Triad Descriptors (CTD) counting the continuous sequences of three amino acids grouped by dipole scale and volume, alongside basic sequence length bounds.	Physicochemical

Table S16: Overview of resampling methods evaluated for regression imbalance correction

Short Name	Full Name	Description
Baseline	Baseline	No resampling applied; data is used as-is with standard scaling.
ADASYN	Adaptive Synthetic Sampling	Generates synthetic minority samples adaptively, placing more near decision boundaries.
Cluster Centroids	Cluster Centroids	Undersamples the majority by replacing clusters of majority samples with their centroids.
CNN	Condensed Nearest Neighbours	Iteratively selects a subset of the majority class that correctly classifies all remaining samples via 1-NN.
ENN	Edited Nearest Neighbours	Removes majority samples whose class label disagrees with the majority vote of their k nearest neighbours.
IHT	Instance Hardness Threshold	Removes instances that are hardest to classify correctly under cross-validated probability estimates.
KNNOR	K-Nearest Neighbour Oversampling for Regression	Generates synthetic samples by interpolating between minority instances and their k nearest neighbours.
NearMiss	NearMiss	Selects majority samples closest to minority samples using one of three distance-based heuristics.
OSS	One-Sided Selection	Combines Tomek link removal with condensed nearest neighbours to clean the majority class boundary.
SMOGN	Synthetic Minority Oversampling with Gaussian Noise	Oversamples rare-target regions using SMOTE-style interpolation combined with Gaussian noise injection.
SMOTER	Synthetic Minority Oversampling for Regression	Applies SMOTE-style interpolation to generate synthetic samples in underrepresented target regions.
Tomek Links	Tomek Links	Removes majority-class members of Tomek link pairs lying on or near the decision boundary.
WERCS	Weighted Relevance-based Combination Strategy	Jointly over- and undersamples using relevance-based weighting of training instances.
WERCS-GN	WERCS with Gaussian Noise	Extends WERCS by adding Gaussian noise to synthetically generated oversampled instances.
Extreme (Tail-Focused) Variants		
EXT ADASYN	Extreme ADASYN	ADASYN variant targeting only the extreme (tail) temperature regions of the target distribution.
EXT Cluster Centroids	Extreme Cluster Centroids	Cluster centroid undersampling applied selectively to the middle temperature range.
EXT CNN	Extreme CNN	Condensed nearest neighbours restricted to undersampling the non-tail temperature region.
EXT ENN	Extreme ENN	Edited nearest neighbours that cleans the middle temperature range while preserving tail instances.
EXT IHT	Extreme IHT	Instance hardness thresholding focused on removing hard-to-predict instances from the middle range.
EXT KNNOR	Extreme KNNOR	KNN-based oversampling restricted to generating synthetic samples in the tail temperature regions.
EXT NearMiss	Extreme NearMiss	NearMiss undersampling applied to the non-tail region to increase relative tail representation.
EXT OSS	Extreme OSS	One-sided selection targeting majority samples in the middle temperature range for removal.

Continued on next page

Short Name	Full Name	Description
EXT Random Over	Extreme Random Oversampling	Random duplication of training instances drawn from the tail temperature regions.
EXT Random Under	Extreme Random Undersampling	Random removal of training instances from the middle temperature range.
EXT SMOGN	Extreme SMOGN	SMOGN oversampling restricted to synthesising new samples in the extreme temperature tails.
EXT SMOTER	Extreme SMOTER	SMOTER interpolation applied exclusively within the tail temperature regions.
EXT TomekLinks	Extreme TomekLinks	Tomek link cleaning applied to the boundary between tail and non-tail temperature regions.
EXT WERCS	Extreme WERCS	Relevance-weighted resampling that boosts tail-region instances while down-weighting the middle range.
EXT WERCS-GN	Extreme WERCS-GN	Extreme WERCS with additional Gaussian noise on oversampled tail instances for diversity.

Table S17: Hyperparameter search space for resampling strategies

Parameter	Description	Tested Values
sampling_strategy	Target ratio of minority to majority samples after resampling.	$\{0.1, 0.2, 0.3, 0.5, 0.7, 1.0, 2.0, 3.0, 5.0\}$
k / k_neighbors	Number of nearest neighbours for synthetic sample generation (ADASYN).	$\{3, 5\}$
n_neighbors	Number of nearest neighbours for cleaning or oversampling.	$\{1, 3, 5, 7\}$
tolerance	Distance tolerance for neighbour-based instance removal (CNN, ENN).	$\{0.0, 0.05, 0.1, 3.0, 5.0, 10.0\}$
noise_std	Standard deviation of Gaussian noise added to synthetic samples (SMOGN, WERCS-GN).	$\{0.01, 0.02, 0.05, 0.1\}$
voting	Strategy for computing cluster centroids (Cluster Centroids).	{soft, hard}
threshold_percentile	Percentile threshold for identifying boundary pairs (TomekLinks).	$\{30, 50, 70, 80, 90, 95\}$
cv	Number of cross-validation folds for Instance Hardness Threshold.	$\{3, 5\}$
rel_thres	Relevance function threshold for determining rare instances (ADASYN).	$\{0.2, 0.25\}$
rel_coef	Relevance function coefficient controlling shape of the relevance curve.	$\{0.5, 1.0, 2.0, 5.0\}$
samp_method	Sampling method for ADASYN (balance vs. extreme mode).	{balance, extreme}
n_seeds_S	Number of seed samples for condensed nearest neighbours (CNN, OSS).	$\{1, 3, 5\}$
version	NearMiss algorithm version controlling the distance heuristic.	$\{1, 2, 3\}$
bins	Number of bins for the relevance function discretisation (SMOGN, SMOTER).	$\{20, 40\}$
critical_percentile	Percentile for identifying the critical zone boundary (OSS).	$\{0.05, 0.1\}$
num_nbrs	Number of neighbours for KNN-based oversampling (KNNOR).	$\{3, 5\}$
proportion_of_minority	Target proportion of minority instances after oversampling (KNNOR).	$\{0.7, 0.9\}$
alpha	Interpolation coefficient for synthetic sample placement (KNNOR).	$\{0.25, 0.5, 0.7\}$

Table S18: Deep learning architectures naming.

Model	Description
Attention MLP	Multi-head self-attention over features combined with a feed-forward MLP.
Baseline MLP	Standard multi-layer perceptron with configurable depth, dropout, and batch normalisation.

Continued on next page

Model	Description
DANets	Deep Abstract Networks that learn to group correlated input features into abstract layers.
FT-Transformer	Feature Tokenizer Transformer that converts each feature into a token processed by a Transformer encoder.
GANDALF	Gated Additive Neural Decision Forest that applies learned feature gates before soft decision trees.
Gated MLP	MLP preceded by per-feature sigmoid gates for soft feature selection.
Hopular	Modern Hopfield network layers that use the training set as associative memory for tabular lookup.
LassoNet	MLP with a linear skip connection and a hierarchy constraint that induces feature-level sparsity.
NODE	Neural Oblivious Decision Ensembles with differentiable oblivious decision trees and entmax splits.
RealMLP	Modern default-tuned MLP with LayerNorm, cosine-annealing schedule, and carefully chosen initialisation.
ResNet MLP	Dense layers with residual (skip) connections enabling deeper MLP training.
RTDL ResNet	Pre-activation residual MLP blocks following the Revisiting Deep Learning for Tabular Data design.
SAINT	Self-Attention and Intersample Attention Transformer operating on both features and samples.
SNN	Self-Normalising Neural Network using SELU activations and AlphaDropout for automatic normalisation.
Sparse MLP	MLP with group-sparse $\ell_{2,1}$ regularisation on the first layer for embedded feature selection.
TabM	Parameter-efficient ensembling of multiple tabular prediction heads within a single backbone.
TabNet	Sequential attentive feature selection with learnable sparsemax masks at each decision step.
TabNet-Inspired	Simplified attention-based sequential feature selector inspired by the TabNet architecture.
TabR	MLP encoder augmented with attention-based retrieval over nearest training neighbours.
Wide & Deep	Combines a wide (linear) component for memorisation with a deep MLP for generalisation.

Table S19: Hyperparameter search spaces for predictive modeling pipelines

Pipeline	Hyperparameter	Search Space
Linear Models		
**LinearRegression**	fit_intercept	{True, False}
	positive	{True, False}
**Ridge**	alpha	$[10^{-6}, 10^{3}]$ (log)
	solver	{auto, svd, cholesky, lsqr, sparse_cg, sag, saga}
	fit_intercept	{True, False}
**ElasticNet**	alpha	$[10^{-5}, 10^{-2}]$ (log)
	l1_ratio	$[0.0, 1.0]$
	fit_intercept	{True, False}
	selection	{cyclic, random}

Continued on next page

Pipeline	Hyperparameter	Search Space
**BayesianRidge**	alpha_1	$[10^{-7}, 10^{-5}]$ (log)
	alpha_2	$[10^{-7}, 10^{-5}]$ (log)
	lambda_1	$[10^{-7}, 10^{-5}]$ (log)
	lambda_2	$[10^{-7}, 10^{-5}]$ (log)
	fit_intercept	{True, False}
**ARDRegression**	alpha_1	$[10^{-7}, 10^{-4}]$ (log)
	alpha_2	$[10^{-7}, 10^{-4}]$ (log)
	lambda_1	$[10^{-7}, 10^{-4}]$ (log)
	lambda_2	$[10^{-7}, 10^{-4}]$ (log)
	fit_intercept	{True, False}
**Huber**	epsilon	$[1.01, 2.0]$
	alpha	$[10^{-5}, 10^{-1}]$ (log)
	fit_intercept	{True, False}
**PassiveAggressive**	C	$[0.001, 10.0]$ (log)
	epsilon	$[0.01, 1.0]$
	loss	{epsilon_insensitive, squared_epsilon_insensitive}
	fit_intercept	{True, False}
**OMP**	n_nonzero_coefs	$[1, 50]$
	fit_intercept	{True, False}
**PLS**	n_components	$[1, 50]$
	scale	{True, False}
**Quantile**	quantile	$\{0.25, 0.5, 0.75\}$
	alpha	$[0.0, 1.0]$
	solver	{highs-ds, highs-ipm, highs}
	fit_intercept	{True, False}
**RANSAC**	min_samples	$[0.1, 0.9]$
	residual_threshold	$[1.0, 10.0]$
Kernel Methods		
**SVR**	C	$[0.01, 1000]$ (log)
	epsilon	$[0.001, 1.0]$ (log)
	kernel	{linear, poly, rbf, sigmoid}
	gamma	{scale, auto}
	degree	$[2, 5]$
**KernelRidge**	alpha	$[0.01, 100]$ (log)
	kernel	{linear, poly, rbf, sigmoid, laplacian}
	gamma	$[0.001, 10.0]$ (log)
	degree	$[2, 5]$
	coef0	$[0.0, 1.0]$
**GPR**	kernel	{RBF, Matern, RationalQuadratic, DotProduct}
	length_scale	$[10^{-3}, 100]$ (log)
	alpha	$[10^{-8}, 10^{-2}]$ (log)
	n_restarts_optimizer	$[0, 10]$
	normalize_y	{True, False}
Scikit-learn Neural Network		
**MLP**	hidden_layer_sizes	{(50,), (100,), (50,50), (100,50), (100,100), (100,50,25)}

Continued on next page

Pipeline	Hyperparameter	Search Space
	activation	{relu, tanh, logistic}
	solver	{adam, sgd, lbfgs}
	alpha	$[10^{-5}, 10^{-1}]$ (log)
	learning_rate	{constant, invscaling, adaptive}
	learning_rate_init	$[10^{-4}, 5 \times 10^{-3}]$ (log)
Decision Trees and Ensembles		
**DecisionTree**	max_depth	$[2, 30]$
	min_samples_split	$[2, 20]$
	min_samples_leaf	$[1, 10]$
	max_features	{sqrt, log2, None}
	splitter	{best, random}
	criterion	{squared_error, friedman_mse, absolute_error, poisson}
**RandomForest / ExtraTrees**	n_estimators	$[50, 500]$
	max_depth	$[3, 30]$
	min_samples_split	$[2, 20]$
	min_samples_leaf	$[1, 10]$
	max_features	{sqrt, log2, None}
	bootstrap	{True, False}
**AdaBoost**	n_estimators	$[50, 500]$
	learning_rate	$[0.001, 2.0]$ (log)
	loss	{linear, square, exponential}
**GradientBoosting**	n_estimators	$[50, 500]$
	learning_rate	$[0.001, 0.3]$ (log)
	max_depth	$[2, 10]$
	min_samples_split	$[2, 20]$
	min_samples_leaf	$[1, 10]$
	subsample	$[0.5, 1.0]$
	max_features	{sqrt, log2, None}
	loss	{squared_error, absolute_error, huber}
**HistGradientBoosting**	max_iter	$[50, 500]$
	learning_rate	$[0.001, 0.3]$ (log)
	max_depth	$[2, 15]$
	min_samples_leaf	$[5, 50]$
	l2_regularization	$[0.0, 10.0]$
	max_bins	$[128, 255]$
	loss	{squared_error, absolute_error}
Gradient Boosting Libraries		
**CatBoost**	iterations	$[50, 500]$
	learning_rate	$[0.001, 0.3]$ (log)
	depth	$[2, 10]$
	l2_leaf_reg	$[1.0, 10.0]$ (log)
	border_count	$[32, 255]$
	random_strength	$[0.0, 10.0]$
	bagging_temperature	$[0.0, 1.0]$
**XGBoost**	n_estimators	$[50, 500]$
	learning_rate	$[0.001, 0.3]$ (log)
	max_depth	$[2, 10]$
	min_child_weight	$[1, 10]$

Continued on next page

Pipeline	Hyperparameter	Search Space
	subsample	$[0.5, 1.0]$
	colsample_bytree	$[0.5, 1.0]$
	gamma	$[0.0, 5.0]$
	reg_alpha	$[0.0, 1.0]$
	reg_lambda	$[0.0, 1.0]$
Bagging Wrappers		
**BaggingSVR**	C	$[0.1, 100]$ (log)
	epsilon	$[0.01, 1.0]$ (log)
	kernel	{rbf}
	gamma	{scale, auto}
**BaggingDecisionTree**	max_depth	$[3, 20]$
	min_samples_split	$[2, 20]$
	min_samples_leaf	$[1, 10]$
	max_features	{sqrt, log2, None}
**BaggingMLP**	hidden_layer_sizes	{(64,), (128,), (64,32), (128,64), (128,64,32)}
	activation	{relu, tanh}
	alpha	$[10^{-5}, 10^{-1}]$ (log)
	learning_rate_init	$[10^{-4}, 10^{-2}]$ (log)
Deep Learning		
**Deep Learning**	learning_rate	{1e-5, 5e-5, 1e-4, 5e-4, 1e-3, 5e-3}
	weight_decay	{0, 1e-5, 1e-4, 1e-3, 1e-2}
	dropout	{0.0, 0.1, 0.2, 0.3, 0.4, 0.5}
	batch_size	{32, 64, 128, 256}
	optimizer	{adam, adamw, radam}
	loss_type	{mse, huber, gaussian_nll, log_cosh}
	activation	{ReLU, GELU, ELU, LeakyReLU, SiLU}
	scheduler	{warmup_cosine, cosine, plateau, onecycle}
	warmup_epochs	$\{0, 3, 5, 10\}$
	gradient_clip	{None, 0.5, 1.0, 5.0}
	early_stopping_patience	$\{10, 15, 20, 30\}$
	scaler_type	{standard, robust, none}

Note: Architecture-specific hyperparameters (e.g., number of layers, hidden dimensions, attention heads) are tuned per model and described in the supplementary materials. For each of BaggingSVR, BaggingDecisionTree, and BaggingMLP, the moderate and Extreme variants share identical base-estimator search spaces. Their bootstrap sampling dynamic ranges are 10× and 1000×, respectively, controlling the preferential sampling of tail observations.

Table S20: Agreement and relative-error metrics on the aligned held-out test set ($n = 997$). Values are point estimates with paired-protein 95% percentile-bootstrap confidence intervals ($B = 10{,}000$).

A. Agreement metrics

Model	OI	R^2 (= NSE)	WI (d_r)
AdventML	**0.779 [0.744, 0.801]**	**0.646 [0.592, 0.691]**	**0.699 [0.673, 0.722]**
AdventML (tail)	0.730 [0.681, 0.761]	0.558 [0.479, 0.621]	0.665 [0.632, 0.693]
AdventML (extreme)	0.718 [0.668, 0.750]	0.536 [0.453, 0.603]	0.649 [0.614, 0.680]
* Seq2Topt	0.673 [0.621, 0.708]	0.455 [0.370, 0.528]	0.629 [0.596, 0.659]
Seq2Topt (retrain)	0.666 [0.625, 0.693]	0.443 [0.378, 0.499]	0.620 [0.594, 0.644]
* SegmentTransformer	0.324 [0.212, 0.400]	-0.190 [-0.385, -0.034]	0.393 [0.333, 0.447]
SegmentTransformer (retrain)	0.756 [0.719, 0.781]	0.606 [0.546, 0.656]	0.695 [0.671, 0.717]
TOMER	0.468 [0.436, 0.487]	0.079 [0.041, 0.118]	0.551 [0.533, 0.567]
* TOMER & Pro-Prime	0.756 [0.713, 0.784]	0.605 [0.537, 0.662]	0.672 [0.641, 0.699]

B. Relative-error metrics

Model	RSE	RSR	Theil U_1	CA
AdventML	**0.354 [0.309, 0.408]**	**0.594 [0.556, 0.639]**	**0.119 [0.112, 0.125]**	**5.963 [5.634, 6.294]**
AdventML (tail)	0.442 [0.379, 0.521]	0.664 [0.615, 0.721]	0.131 [0.124, 0.139]	6.674 [6.310, 7.058]
AdventML (extreme)	0.464 [0.397, 0.547]	0.681 [0.630, 0.739]	0.134 [0.127, 0.142]	6.903 [6.539, 7.278]
* Seq2Topt	0.545 [0.472, 0.630]	0.738 [0.687, 0.794]	0.144 [0.136, 0.152]	7.423 [7.003, 7.849]
Seq2Topt (retrain)	0.557 [0.501, 0.622]	0.746 [0.708, 0.788]	0.151 [0.144, 0.159]	7.540 [7.140, 7.946]
* SegmentTransformer	1.190 [1.034, 1.385]	1.090 [1.016, 1.176]	0.193 [0.184, 0.201]	11.575 [11.092, 12.077]
SegmentTransformer (retrain)	0.394 [0.344, 0.454]	0.627 [0.586, 0.674]	0.127 [0.119, 0.135]	6.195 [5.805, 6.591]
TOMER	0.921 [0.882, 0.959]	0.959 [0.939, 0.979]	0.198 [0.187, 0.209]	9.437 [8.828, 10.044]
* TOMER & Pro-Prime	0.395 [0.338, 0.463]	0.628 [0.581, 0.680]	0.120 [0.113, 0.128]	6.399 [6.066, 6.746]

OI denotes the overall index, combining range-normalised RMSE and relative squared error. WI denotes Willmott's refined index of agreement (d_r), comparing absolute prediction errors with the variability of the observations. NSE denotes Nash-Sutcliffe efficiency, equivalent here to R^2, RSE denotes relative squared error, and RSR denotes the ratio of RMSE to the observed standard deviation. Theil U_1 measures prediction error relative to the magnitudes of the observed and predicted temperatures. CA denotes combined accuracy, a descriptive composite of RMSE, MAE, and RSE. Higher OI, NSE, and WI indicate better agreement, while lower RSE, RSR, Theil U_1, and CA indicate smaller error. AdventML achieved the highest OI (0.779) and WI (0.699), together with the lowest Theil U_1 (0.119) and CA (5.963), giving the best point estimates across these measures. WI was close to that of retrained SegmentTransformer (0.695), and Theil U_1 was close to that of TOMER & Pro-Prime (0.120). Bold marks the best point estimate for each metric. An asterisk denotes overlap between a model's original training data and the test set. Notice that all metrics are calculated over the complete held-out test set distribution and, as such, do not specifically reflect performance at the temperature extremes.

Pseudocode for Resampling Strategies

The following pseudocode describes the resampling algorithms implemented in this work for imbalanced regression tasks. All algorithms operate on a dataset $D(X, y)$ with features X and continuous target values y.

Oversampling Methods

Algorithm 1 ADASYN Regressor (Adaptive Synthetic Sampling)

Require: $D(X, y)$ - dataset with features X and continuous target y
Require: k - number of nearest neighbors
Require: t_R - relevance threshold (quantile on $|y - \text{median}(y)|$)
Require: c - relevance coefficient (multiplier for synthetic samples)
Require: *method* - sampling method: 'balance' or 'extreme'
Ensure: *newD* - resampled dataset

$d_i \leftarrow |y_i - \text{median}(y)|$ for all instances
$\tau \leftarrow \text{quantile}(d, t_R)$
extremeD $\leftarrow$ instances in D where $d_i \geq \tau$
nonExtremeD $\leftarrow$ instances in D where $d_i < \tau$
if *method* = 'balance' **then**
    $G \leftarrow (|\mathit{nonExtremeD}| - |\mathit{extremeD}|) \times c$
else
    $G \leftarrow |\mathit{extremeD}| \times c$
end if
nns $\leftarrow$ get k-nearest neighbors for all instances in *extremeD*
for each instance x_i in *extremeD* **do**
    $r_i \leftarrow$ fraction of neighbors that are non-extreme
end for
$\hat{r}_i \leftarrow r_i / \sum r_i$ ▷ normalize
$g_i \leftarrow \text{round}(\hat{r}_i \times G)$ ▷ allocate synthetic samples per instance
syntheticD $\leftarrow \emptyset$
for each instance x_i in *extremeD* with allocation g_i **do**
    for $j \leftarrow 1$ to g_i **do**
        $x_{nb} \leftarrow$ random neighbor from $nns[i]$
        $\lambda \leftarrow \text{random}(0, 1)$
        $x_{syn} \leftarrow x_i + \lambda \times (x_{nb} - x_i)$
        $y_{syn} \leftarrow y_i + \lambda \times (y_{nb} - y_i)$
        $\mathit{syntheticD} \leftarrow \mathit{syntheticD} \cup \{(x_{syn}, y_{syn})\}$
    end for
end for
return $D \cup \mathit{syntheticD}$

Undersampling Methods

Extreme-Only Methods

The following methods are implemented only with fixed tail thresholds, as they are simple sampling strategies that benefit from domain-specific region definitions.

Extreme Variants of Adaptive Methods

The extreme variants of all above adaptive algorithms use **fixed tail thresholds** instead of adaptive/histogram-based detection of rare regions. Specifically:

Algorithm 2 SMOTER Regressor (SMOTE for Regression)

Require: $D(X, y)$ - dataset with features X and continuous target y
Require: s - sampling strategy (number or fraction of synthetic samples)
Require: k - number of nearest neighbors
Require: b - number of bins (default: $\sqrt{n}$)
Ensure: $newD$ - resampled dataset

$hist, edges \leftarrow$ histogram(y, b)
$avg \leftarrow$ mean$(hist)$
$rareBins \leftarrow$ bins where $hist[i] < avg$
$rareD \leftarrow$ instances in D falling in $rareBins$
$m \leftarrow |rareD|$
if s is float **then**
    $n_{synth} \leftarrow \lfloor s \times m \rfloor$
else
    $n_{synth} \leftarrow s$
end if
$nns \leftarrow$ get k-nearest neighbors for all instances in $rareD$
$syntheticD \leftarrow \emptyset$
while $|syntheticD| < n_{synth}$ **do**
    $i \leftarrow$ random index in $rareD$
    $j \leftarrow$ random neighbor from $nns[i]$
    $u \leftarrow$ random$(0, 1)$
    $x_{syn} \leftarrow x_i + u \times (x_j - x_i)$
    $y_{syn} \leftarrow y_i + u \times (y_j - y_i)$
    $syntheticD \leftarrow syntheticD \cup \{(x_{syn}, y_{syn})\}$
end while
return $D \cup syntheticD$

Algorithm 3 SMOGN Regressor (SMOTE with Gaussian Noise)

Require: $D(X, y)$ - dataset with features X and continuous target y
Require: s - sampling strategy (number or fraction of synthetic samples)
Require: k - number of nearest neighbors
Require: b - number of bins (default: $\sqrt{n}$)
Require: σ - standard deviation for Gaussian noise
Ensure: $newD$ - resampled dataset

$hist, edges \leftarrow \text{histogram}(y, b)$
$avg \leftarrow \text{mean}(hist)$
$rareBins \leftarrow$ bins where $hist[i] < avg$
$rareD \leftarrow$ instances in D falling in $rareBins$
$m \leftarrow |rareD|$
if s is float **then**
    $n_{synth} \leftarrow \lfloor s \times m \rfloor$
else
    $n_{synth} \leftarrow s$
end if
$nns \leftarrow$ get k-nearest neighbors for all instances in $rareD$
$syntheticD \leftarrow \emptyset$
while $|syntheticD| < n_{synth}$ **do**
    $i \leftarrow$ random index in $rareD$
    $j \leftarrow$ random neighbor from $nns[i]$
    $u \leftarrow \text{random}(0, 1)$
    $x_{syn} \leftarrow x_i + u \times (x_j - x_i) + \mathcal{N}(0, \sigma)$
    $y_{syn} \leftarrow y_i + u \times (y_j - y_i) + \mathcal{N}(0, \sigma)$
    $syntheticD \leftarrow syntheticD \cup \{(x_{syn}, y_{syn})\}$
end while
return $D \cup syntheticD$

Algorithm 4 WERCS Regressor (Weighted Relevance Combination Sampling)

Require: $D(X, y)$ - dataset with features X and continuous target y
Require: s - sampling strategy (number or fraction of synthetic samples)
Require: k - number of nearest neighbors
Ensure: $newD$ - resampled dataset

$n \leftarrow |D|$
if s is float **then**
  $n_{synth} \leftarrow \lfloor s \times n \rfloor$
else
  $n_{synth} \leftarrow s$
end if
$med \leftarrow \text{median}(y)$
$w_i \leftarrow |y_i - med|$ for all instances ▷ weight by deviation from median
$\hat{w}_i \leftarrow w_i / \sum w_i$ ▷ normalize weights
$c_i \leftarrow \lfloor \hat{w}_i \times n_{synth} \rfloor$ ▷ allocate counts per instance
Adjust c_i to ensure $\sum c_i = n_{synth}$
$nns \leftarrow$ get k-nearest neighbors for all instances in D
$syntheticD \leftarrow \emptyset$
for each instance x_i in D with allocation c_i **do**
  for $j \leftarrow 1$ to c_i **do**
    $x_{nb} \leftarrow$ random neighbor from $nns[i]$
    $u \leftarrow \text{random}(0, 1)$
    $x_{syn} \leftarrow x_i + u \times (x_{nb} - x_i)$
    $y_{syn} \leftarrow y_i + u \times (y_{nb} - y_i)$
    $syntheticD \leftarrow syntheticD \cup \{(x_{syn}, y_{syn})\}$
  end for
end for
return $D \cup syntheticD$

Algorithm 5 WERCS-GN Regressor (WERCS with Gaussian Noise)

Require: $D(X, y)$ - dataset with features X and continuous target y
Require: s - sampling strategy (number or fraction of synthetic samples)
Require: k - number of nearest neighbors
Require: σ - standard deviation for Gaussian noise
Ensure: $newD$ - resampled dataset

$n \leftarrow |D|$
if s is float **then**
  $n_{synth} \leftarrow \lfloor s \times n \rfloor$
else
  $n_{synth} \leftarrow s$
end if
$med \leftarrow \text{median}(y)$
$w_i \leftarrow |y_i - med|$ for all instances
$\hat{w}_i \leftarrow w_i / \sum w_i$
$c_i \leftarrow \lfloor \hat{w}_i \times n_{synth} \rfloor$
Adjust c_i to ensure $\sum c_i = n_{synth}$
$nns \leftarrow$ get k-nearest neighbors for all instances in D
$syntheticD \leftarrow \emptyset$
for each instance x_i in D with allocation c_i **do**
  for $j \leftarrow 1$ to c_i **do**
    $x_{nb} \leftarrow$ random neighbor from $nns[i]$
    $u \leftarrow \text{random}(0, 1)$
    $x_{syn} \leftarrow x_i + u \times (x_{nb} - x_i) + \mathcal{N}(0, \sigma)$
    $y_{syn} \leftarrow y_i + u \times (y_{nb} - y_i) + \mathcal{N}(0, \sigma)$
    $syntheticD \leftarrow syntheticD \cup \{(x_{syn}, y_{syn})\}$
  end for
end for
return $D \cup syntheticD$

Algorithm 6 KNNOR Regressor (K-Nearest Neighbors Oversampling for Regression)

Require: $D(X, y)$ - dataset with features X and continuous target y
Require: k - number of nearest neighbors
Require: p - proportion of minority for threshold determination
Require: α - maximum interpolation factor
Require: b - number of bins (default: $\sqrt{n}$)
Require: t - target count per bin (default: mean bin frequency)
Ensure: $newD$ - resampled dataset

$hist, edges \leftarrow \text{histogram}(y, b)$
if $t = \text{None}$ **then**
$t \leftarrow \text{mean}(hist)$
end if
$syntheticD \leftarrow \emptyset$
for each bin i where $hist[i] < t$ **do**
$D_{bin} \leftarrow$ instances in D falling in bin i
$toAdd \leftarrow t - |D_{bin}|$
Compute k-th neighbor distances for all instances in D_{bin}
$\tau \leftarrow$ threshold at p-th percentile of distances
while $toAdd > 0$ **do**
for each x_i in D_{bin} where k-th neighbor distance $\leq \tau$ **do**
$x_{syn} \leftarrow x_i$
for $j \leftarrow 1$ to k **do**
$x_{nb} \leftarrow j$-th neighbor of x_i
$\lambda \leftarrow \text{random}(0, \alpha)$
$x_{syn} \leftarrow x_{syn} + \lambda \times (x_{nb} - x_i)$
end for
$y_{syn} \leftarrow$ inverse-distance weighted average of neighbor y values
$syntheticD \leftarrow syntheticD \cup \{(x_{syn}, y_{syn})\}$
$toAdd \leftarrow toAdd - 1$
if $toAdd = 0$ **then**
break
end if
end for
end while
end for
return $D \cup syntheticD$

Algorithm 7 Cluster Centroids Regressor

Require: $D(X, y)$ - dataset with features X and continuous target y
Require: s - sampling strategy (desired number or fraction of samples)
Require: *voting* - strategy: 'hard' or 'soft'
Ensure: *newD* - resampled dataset

$n \leftarrow |D|$
if s is float **then**
  $n_{samples} \leftarrow \lfloor s \times n \rfloor$
else
  $n_{samples} \leftarrow s$
end if
Fit KMeans clustering with $n_{clusters} = n_{samples}$
centers $\leftarrow$ cluster centroids
if *voting* = 'hard' **then**
  Find nearest original sample to each centroid
  $X_{res} \leftarrow$ nearest samples' features
  $y_{res} \leftarrow$ nearest samples' targets
else ▷ soft voting
  $X_{res} \leftarrow$ *centers*
  $y_{res}[i] \leftarrow \text{mean}(y_j)$ for all j assigned to cluster i
end if
return (X_{res}, y_{res})

Algorithm 8 CNN Regressor (Condensed Nearest Neighbour)

Require: $D(X, y)$ - dataset with features X and continuous target y
Require: s - sampling strategy (desired number or fraction of samples)
Require: k - number of neighbors for KNN regressor
Require: n_{seeds} - number of initial seed samples
Require: ϵ - tolerance threshold for prediction error
Ensure: *newD* - resampled dataset

$n \leftarrow |D|$
if s is float **then**
  $n_{desired} \leftarrow \lfloor s \times n \rfloor$
else
  $n_{desired} \leftarrow s$
end if
$C \leftarrow$ randomly select n_{seeds} instances from D ▷ condensed set
changed $\leftarrow$ True
while *changed* **do**
  changed $\leftarrow$ False
  Train KNN regressor on C
  for each instance (x_i, y_i) in $D \setminus C$ **do**
    $\hat{y}_i \leftarrow$ KNN prediction for x_i
    if $|y_i - \hat{y}_i| > \epsilon$ **then**
      $C \leftarrow C \cup \{(x_i, y_i)\}$
      changed $\leftarrow$ True
    end if
  end for
end while
if $|C| > n_{desired}$ **then**
  $C \leftarrow$ random subset of C with $n_{desired}$ instances
end if
return C

Algorithm 9 ENN Regressor (Edited Nearest Neighbours)

Require: $D(X, y)$ - dataset with features X and continuous target y
Require: s - sampling strategy (desired number or fraction of samples)
Require: k - number of neighbors
Require: ϵ - tolerance threshold for prediction error
Ensure: *newD* - resampled dataset

$n \leftarrow |D|$
if s is float **then**
    $n_{desired} \leftarrow \lfloor s \times n \rfloor$
else
    $n_{desired} \leftarrow s$
end if
Train KNN regressor on D
for each instance (x_i, y_i) in D **do**
    neighbors $\leftarrow k$ nearest neighbors of x_i (excluding self)
    $\hat{y}_i \leftarrow \text{mean}(y_j)$ for j in *neighbors*
    $e_i \leftarrow |y_i - \hat{y}_i|$
end for
keepIndices $\leftarrow$ indices where $e_i \leq \epsilon$
if $|keepIndices| > n_{desired}$ **then**
    keepIndices $\leftarrow$ random subset with $n_{desired}$ indices
end if
return instances in D at *keepIndices*

Algorithm 10 IHT Regressor (Instance Hardness Threshold)

Require: $D(X, y)$ - dataset with features X and continuous target y
Require: s - sampling strategy (desired number or fraction of samples)
Require: *cv* - number of cross-validation folds
Require: *estimator* - regression model (default: RandomForest)
Ensure: *newD* - resampled dataset

$n \leftarrow |D|$
if s is float **then**
    $n_{desired} \leftarrow \lfloor s \times n \rfloor$
else
    $n_{desired} \leftarrow s$
end if
$\hat{y} \leftarrow$ cross-validated predictions using *estimator* with *cv* folds
$e_i \leftarrow |y_i - \hat{y}_i|$ for all instances ▷ instance hardness
sortedIndices $\leftarrow \text{argsort}(e)$ ▷ sort by ascending error
selected $\leftarrow$ *sortedIndices*$[1 : n_{desired}]$ ▷ keep easiest instances
return instances in D at *selected*

Algorithm 11 NearMiss Regressor

Require: $D(X, y)$ - dataset with features X and continuous target y
Require: s - sampling strategy (number or fraction of dense-region samples to retain)
Require: *version* - NearMiss version: 1, 2, or 3
Require: k - number of neighbors
Require: b - number of bins (default: $\sqrt{n}$)
Ensure: *newD* - resampled dataset

hist, *edges* $\leftarrow$ histogram(y, b)
avg $\leftarrow$ mean(*hist*)
sparseBins $\leftarrow$ bins where *hist*[*i*] $\leq$ *avg*
denseBins $\leftarrow$ bins where *hist*[*i*] $>$ *avg*
sparseD $\leftarrow$ instances falling in *sparseBins*
denseD $\leftarrow$ instances falling in *denseBins*
if s is float **then**
    $n_{keep} \leftarrow \lfloor s \times |denseD| \rfloor$
else
    $n_{keep} \leftarrow s$
end if
if *version* $= 1$ **then**
    For each x_i in *denseD*: compute mean distance to k nearest sparse neighbors
    selected $\leftarrow n_{keep}$ instances with smallest mean distance
else if *version* $= 2$ **then**
    For each x_i in *denseD*: compute mean distance to all sparse instances
    selected $\leftarrow n_{keep}$ instances with smallest mean distance
else ▷ version 3
    For each x_i in *sparseD*: find k nearest dense neighbors
    candidates $\leftarrow$ unique dense neighbors found
    For each x_i in *candidates*: compute mean distance to k nearest sparse neighbors
    selected $\leftarrow n_{keep}$ candidates with largest mean distance
end if
return instances in *denseD* at *selected*

Algorithm 12 OSS Regressor (One-Sided Selection)

Require: $D(X, y)$ - dataset with features X and continuous target y
Require: s - sampling strategy (number or fraction of non-critical samples to retain)
Require: p - critical percentile (defines lower and upper tails)
Require: k - number of neighbors for KNN
Require: n_{seeds} - number of initial seeds from non-critical region
Ensure: $newD$ - resampled dataset

$low \leftarrow$ percentile$(y, p \times 100)$
$high \leftarrow$ percentile$(y, 100 - p \times 100)$
$criticalD \leftarrow$ instances where $y \leq low$ or $y \geq high$
$nonCritD \leftarrow$ instances where $low < y < high$
if s is float **then**
    $n_{keep} \leftarrow \lfloor s \times |nonCritD| \rfloor$
else
    $n_{keep} \leftarrow s$
end if
$seeds \leftarrow$ random selection of n_{seeds} instances from $nonCritD$
$C \leftarrow criticalD \cup seeds$ ▷ initial condensed set
Train KNN regressor on C
$remaining \leftarrow nonCritD \setminus seeds$
for each (x_i, y_i) in $remaining$ **do**
    $e_i \leftarrow |y_i -$ KNN.predict$(x_i)|$
end for
$selectedNonCrit \leftarrow n_{keep} - n_{seeds}$ instances with largest e_i
return $criticalD \cup seeds \cup selectedNonCrit$

Algorithm 13 Tomek Links Regressor

Require: $D(X, y)$ - dataset with features X and continuous target y
Require: p - threshold percentile for target difference
Ensure: $newD$ - resampled dataset

$nn \leftarrow$ 1-nearest neighbor for all instances in D
$mutualPairs \leftarrow \emptyset$
for each instance i in D **do**
    $j \leftarrow$ nearest neighbor of i
    if nearest neighbor of j is i **then** ▷ mutual nearest neighbors
        $mutualPairs \leftarrow mutualPairs \cup \{(i, j)\}$ where $i < j$
    end if
end for
$diffs \leftarrow |y_i - y_j|$ for all (i, j) in $mutualPairs$
$\tau \leftarrow$ percentile$(diffs, p)$
$toRemove \leftarrow \emptyset$
for each (i, j, d) in $(mutualPairs, diffs)$ **do**
    if $d \geq \tau$ **then** ▷ Tomek link with large target discrepancy
        $toRemove \leftarrow toRemove \cup \{i, j\}$
    end if
end for
return $D \setminus toRemove$

Algorithm 14 Extreme Random Oversampling Regressor

Require: $D(X, y)$ - dataset with features X and continuous target y
Require: s - sampling strategy (number or fraction of samples to duplicate)
Require: $\tau_{low} = 25$ - lower tail threshold
Require: $\tau_{high} = 60$ - upper tail threshold
Ensure: $newD$ - resampled dataset

$tailD \leftarrow$ instances where $y < \tau_{low}$ or $y > \tau_{high}$
$m \leftarrow |tailD|$
if $m = 0$ **then**
    return D
end if
if s is float **then**
    $n_{synth} \leftarrow \lfloor s \times m \rfloor$
else
    $n_{synth} \leftarrow s$
end if
$duplicatedIdx \leftarrow$ randomly sample n_{synth} indices from $tailD$ with replacement
$X_{syn} \leftarrow X[duplicatedIdx]$
$y_{syn} \leftarrow y[duplicatedIdx]$
return $D \cup (X_{syn}, y_{syn})$

Algorithm 15 Extreme Random Undersampling Regressor

Require: $D(X, y)$ - dataset with features X and continuous target y
Require: s - sampling strategy (number or fraction of middle samples to retain)
Require: $\tau_{low} = 25$ - lower tail threshold
Require: $\tau_{high} = 60$ - upper tail threshold
Ensure: $newD$ - resampled dataset

$tailD \leftarrow$ instances where $y < \tau_{low}$ or $y > \tau_{high}$
$middleD \leftarrow$ instances where $\tau_{low} \leq y \leq \tau_{high}$
$m \leftarrow |middleD|$
if $m = 0$ **then**
    return D
end if
if s is float **then**
    $n_{keep} \leftarrow \lfloor s \times m \rfloor$
else
    $n_{keep} \leftarrow s$
end if
$keepIdx \leftarrow$ randomly sample n_{keep} indices from $middleD$ without replacement
$middleD_{res} \leftarrow$ instances at $keepIdx$
return $tailD \cup middleD_{res}$ ▷ all tail samples preserved

- **Tail region**: instances where $y < 25$ or $y > 60$
- **Middle region**: instances where $25 \leq y \leq 60$

Algorithm 16 Extreme Variant Modification

Require: $D(X, y)$ - dataset with features X and continuous target y
Require: $\tau_{low} = 25$ - lower tail threshold
Require: $\tau_{high} = 60$ - upper tail threshold
Ensure: Region identification for extreme variants
1: *tailD* $\leftarrow$ instances where $y < \tau_{low}$ or $y > \tau_{high}$
2: *middleD* $\leftarrow$ instances where $\tau_{low} \leq y \leq \tau_{high}$
3: **For oversampling methods** (ADASYN, SMOTER, SMOGN, WERCS, WERCS-GN, KNNOR):
4: Generate synthetic samples **only from** *tailD*
5: All original samples in D are preserved
6: **For undersampling methods** (ClusterCentroids, CNN, ENN, IHT, NearMiss, OSS, TomekLinks):
7: Apply undersampling **only to** *middleD*
8: All samples in *tailD* are preserved unchanged

Key differences between standard and extreme variants:

1. **Standard variants**: Use adaptive thresholds based on:
   - Histogram binning (SMOTER, SMOGN, KNNOR, NearMiss)
   - Quantile-based relevance thresholds (ADASYN)
   - Percentile-based critical regions (OSS)
   - Prediction error thresholds (CNN, ENN, IHT)
   - Target discrepancy percentiles (TomekLinks)
   - Deviation from median (WERCS, WERCS-GN)
2. **Extreme variants**: Use fixed domain-specific thresholds ($y < 25$ or $y > 60$) to identify samples requiring special handling, ensuring that tail regions are always prioritized regardless of the dataset's distribution.
